# Phytochemical characterization, total phenolic and flavonoid content, antioxidant capacity, enzymatic profiling, and cytotoxicity of *Bidens pilosa* and *Croton* sp. from Colombia for applications in skin health

**DOI:** 10.1371/journal.pone.0340869

**Published:** 2026-01-09

**Authors:** Patricia Quintero-Rincón, Karina Caballero-Gallardo, Elkin Galeano, Oscar Flórez-Acosta

**Affiliations:** 1 Research Group Design and Formulation of Medicines, Cosmetics, and Related, Faculty of Pharmaceutical and Food Sciences, Universidad de Antioquia, Medellin, Colombia; 2 Functional Toxicology Group. School of Pharmaceutical Sciences, Zaragocilla Campus, University of Cartagena, Cartagena, Colombia; 3 Environmental and Computational Chemistry Group, School of Pharmaceutical Sciences, Zaragocilla Campus, University of Cartagena, Cartagena, Colombia; 4 Bioactive Substances Research Group, Faculty of Pharmaceutical and Food Sciences, Universidad de Antioquia, Medellin, Colombia; Universidad San Francisco de Quito - Campus Cumbaya: Universidad San Francisco de Quito, ECUADOR

## Abstract

This study investigated the chemical and biological potential of *Bidens pilosa* and *Croton* sp., plants from megadiverse ecosystems in Colombia, collected in Santander de Quilichao (Cauca) and San Basilio de Palenque (Bolivar). The chemical profile was analyzed by UHPLC-ESI-Orbitrap-HRMS, and the total phenolic and flavonoid content was quantified using colorimetric methods. Antioxidant capacity was assessed using methods that evaluate reducing power and electron transfer mechanisms. The inhibition of key enzymes in skin aging, such as tyrosinase, hyaluronidase, and collagenase, was evaluated, as well as cytotoxicity in keratinocytes and human melanoma cells. Chemical characterization revealed distinctive phytochemical profiles: *B. pilosa* contained 21.1 mg GAE/g DT of phenolics and 64.6 mg RE/g DT of flavonoids, dominated by *p*-coumaric acid and rosmarinic acid, while *Croton* sp. exhibited higher levels of phenolics (169.4 mg GAE/g DT) and 54.1 mg RE/g DT of flavonoids, highlighting rosmarinic acid, *p*-coumaric acid and quercetin. Both extracts showed significant antioxidant capacity and enzyme modulation, including moderate collagenase inhibition (53.9–55.0%), high hyaluronidase inhibition (64.5–76.5%), and low tyrosinase inhibition (11.1–12.7%), suggesting protection of extracellular matrix and hyaluronic acid during skin aging. Sun protection factor was limited (SPF: 14.5 for *B. pilosa* and 11.6 for *Croton* sp.), with low ultraviolet absorption, consistent with low antityrosinase activity. Cytotoxicity assays demonstrated that *B. pilosa* was not toxic to HaCaT keratinocytes (IC₅₀ > 500 µg/mL) and displayed antimelanoma activity on A375 cells (IC₅₀ = 398.6 µg/mL), whereas *Croton* sp. showed moderate selectivity towards melanoma cells (IC₅₀ HaCaT = 329.5 µg/mL; IC₅₀ A375 = 189.0 µg/mL). The results suggest that both plants have potential in dermatological applications such as anti-melanoma agents, antioxidants, and modulators of skin aging enzymes, although highlight the importance of improving strategies to maximize their efficacy and safety.

## Introduction

Colombia, recognized as one of the most megadiverse countries in the world, is home to contrasting ecosystems ranging from humid forests to arid zones, where flora has developed unique resilience strategies [1,2]. This diversity constitutes a valuable reservoir of bioactive compounds with potential applications in health and cosmetics, as well as possessing profound ethnocultural value based on ancestral knowledge associated with medicinal plants [3–7]. Research in different Colombian regions have identified species with properties relevant to skin care, including antioxidant, anti-aging, anti-inflammatory, antimicrobial, and photoprotective activity, underscoring the importance of promoting the systematic and sustainable study of these local species [8–14].

In this research, *Bidens pilosa* and *Croton* sp. were selected for their scientific relevance in contrasting Colombian contexts. *B. pilosa* collected in Santander de Quilichao, stands out for its ethnomedicinal use and pharmacological properties, positioning itself as a strategic candidate for bioprospecting of bioactive metabolites from flora in landscapes influenced by mining and agricultural practices. On the other hand, *Croton* sp., collected in San Basilio de Palenque, a site declared Intangible Cultural Heritage of UNESCO, represents the integration of ancestral knowledge about the use of plants, highlighting its ethnopharmacological importance [11].

*B. pilosa* L. (Asteraceae) is an erect annual herb native to tropical and subtropical America and widely distributed in Colombia. It occurs in the Andean, Caribbean, and Pacific regions, from sea level to 3100 m a.s.l., and inhabits diverse environments such as forests, savannas, shrublands, grasslands, wetlands, and disturbed areas. The species is characterized by yellow disk florets, white ray florets, and strongly adherent fruits that favor dispersal. Known by vernacular names including Black Jack, Abujua (Cubeo), Cadillo, Pacunga, and Papunga, it is reported in several Colombian departments and classified as potentially of Least Concern (LC) (https://powo.science.kew.org/taxon/urn:lsid:ipni.org:names:32564-2/general-information accessed September 24, 2025). *B. pilosa* has been traditionally used for diverse medicinal purposes. Its extracts and leaves treat infections, ocular pain, abdominal discomfort, burns, and wounds. It is also valued as a choleretic, diuretic, antitumor, and antidiabetic agent, with applications in respiratory, glandular, and urinary disorders [15]. From a biofunctional perspective, the species is recognized for its antioxidant, anti-inflammatory, antimicrobial, immunomodulatory, and anticancer properties [16–19].

*Croton* sp. (Euphorbiaceae) is a shrub characterized by alternate leaves, small inflorescences, and capsular fruits. Due to limitations in morphological identification, it has been classified only at the genus level. Ethnomedicinally, local communities and indigenous populations use the leaves and stems of *Croton* sp. for treating fever, wounds, malaria, minor inflammatory conditions, and gastrointestinal discomfort [20,21]. In different studies, preliminary biological assays on hydroalcoholic extracts revealed notable antioxidant, anti-inflammatory, immunomodulatory, antiparasitic, and antimicrobial activities, suggesting bioactive potential of genus. Although these findings are not specific to the specimen studied, they highlight the importance of further taxonomic identification and thorough phytochemical analysis to fully characterize its pharmacological properties [22,23].

This study aimed to quantify the total phenolic and flavonoid content and identify the main metabolites in hydroalcoholic extracts of *B. pilosa* and *Croton* sp. using UHPLC-ESI-Orbitrap-HRMS. Antioxidant capacity was assessed through ferric reducing antioxidant power (FRAP) and 2,2’-azino-bis(3-ethylbenzothiazoline-6-sulfonic acid (ABTS) assays, while inhibitory activity against skin-aging-related enzymes (collagenase, hyaluronidase, and tyrosinase) was also determined. Photoprotective properties were evaluated using UV spectrophotometric parameters and compared with a commercial sunscreen. *In vitro* assays in HaCaT keratinocytes and A375 melanoma cells further assessed cytotoxicity and selectivity indices. Together, these approaches provided a comprehensive overview of the functional properties of both species, highlighting the pharmacological potential of *B. pilosa* in antimelanoma applications and the distinctive antioxidant capacity of *Croton* sp.

## Results

### Chemical analysis

Chemical analysis of hydroalcoholic extracts of *B. pilosa* and *Croton* sp. showed distinct phenolic and flavonoid profiles. *B. pilosa* presented 21.1 mg of gallic acid equivalent per g of dry tissue (mg GAE/g DT) of phenolics and 64.6 mg of rutin per g of dry tissue (mg RE/g DT) of flavonoids, while *Croton* sp. exhibited a higher phenolic content (169.4 mg GAE/g DT) and 54.1 mg RE/g DT of flavonoids (Table 1). Furthermore, twenty phytochemicals were identified and quantified using UHPLC-ESI±Orbitrap-HRMS, and the detailed results are presented in Supplementary S1 Table. In *B. pilosa*, most compounds were below the limit of quantification (LQ < 0.4 mg/kg), except for *p*-coumaric acid (0.7 mg/kg) and rosmarinic acid (67.2 mg/kg) (Table 1). In *Croton* sp., higher levels of *p*-coumaric acid (2.9 mg/kg), rosmarinic acid (55.9 mg/kg), and quercetin (20.4 mg/kg) were detected (Table 1), while the rest of the compounds remained below the LQ. These results show a differentiated phytochemical profile, highlighting the abundance of rosmarinic acid and the specific presence of quercetin in *Croton* sp., although exhaustive chemical characterizations are required to fully understand the compositional richness of the evaluated extracts.

**Table 1:** Total phenolic and flavonoid contents, and major compounds by UHPLC-ESI-Orbitrap-HRMS.

Hydroalcoholic extract	Total phenolics (mg GAE/g of dry tissue)*	Total flavonoids (mg RE/g of dry tissue)*	Major compounds (mg/kg of dry sample)
*B. pilosa*	21.1 ± 0.1	64.6 ± 0.3	*p*-Coumaric acid (0.7) and rosmarinic acid (67.2)
*Croton* sp.	169.4 ± 0.1	54.1 ± 0.7	*p*-Coumaric acid (2.9), rosmarinic acid (55.9), and quercetin (20.4)

* The results are presented as mean ± standard deviation

### Biological activity

#### Antioxidant capacity and enzymatic inhibition.

Hydroalcoholic extracts of *B. pilosa* and *Croton* sp., showed significant antioxidant capacity and inhibitory activity against enzymes related to skin aging (Table 2). In *in vitro* assays, *B. pilosa* showed a reducing power of 14,999.1 μmol TE/100 g DT in FRAP, which evaluates the extract’s ability to reduce ferric ions (Fe³⁺) to ferrous ions (Fe²⁺). In addition, it exhibited an ABTS• ⁺ radical scavenging capacity of 25,183.4 μmol TE/100 g in the TEAC-ABTS assay, which measures the neutralization of the ABTS• ⁺ radical and enables the assessment of both lipophilic and hydrophilic antioxidants, providing an overall indication of antioxidant potential. For its part, *Croton* sp. exhibited a slightly lower value in FRAP (12,902.9 μmol TE/100 g DT), but higher in TEAC-ABTS (49,533.3 μmol TE/100 g DT), evidencing a differentiated antioxidant profile, with greater efficacy in capturing free radicals and reactive oxygen species (ROS). In the enzymatic models, both extracts exhibited moderate collagenase inhibition (53.9–55.0%), high hyaluronidase inhibition (64.5–76.5%) and low tyrosinase inhibition (11.1–12.7%), evidencing their potential to modulate processes associated with the degradation of the extracellular matrix and the degradation of hyaluronic acid during aging, with less effect on skin depigmentation.

**Table 2:** Assessment of antioxidant capacity and skin aging–related enzyme activity.

Hydroalcoholic extract	FRAP (µmol TE/100 g)	TEAC-ABTS (µmol TE/100 g)	Collagenase inhibition (%)	Hyaluronidase inhibition (%)	Tyrosinase inhibition (%)
*B. pilosa*	14,999.1 ± 200.6	25,183.4 ± 7508.9	53.9 ± 1.8	76.5 ± 2.3	11.08 ± 3.6
*Croton* sp.	12,902.9 ± 434.2	49,533.3 ± 3467.4	55.0 ± 1.1	64.5 ± 1.3	12.67 ± 4.7

Results expressed as mean ± standard deviation (n = 3). Enzymatic assays performed with a final extract concentration of 250 µg/mL. Standards: kojic acid (100 µM) for tyrosinase and EGCG (5 and 10 µM) for collagenase and hyaluronidase. Kojic acid inhibited tyrosinase by 99.4%, while EGCG inhibited collagenase and hyaluronidase by 91.4% and 93.8%, respectively

#### Photoprotective potential.

The hydroalcoholic extracts of *B. pilosa* and *Croton* sp. showed a slight photoprotective potential according to the UV indices determined by spectrophotometric methods (Table 3). *B. pilosa* presented an SPF of 14.5 and *Croton* sp. of 11.6, values considerably lower than that of the reference sunscreen (SPF 53.2). Both extracts showed λc close to 351 nm and a UVA/UVB ratio of 2.0, with low erythema and pigmentation transmissions but higher than the control. This profile suggests that, although they have some UV radiation absorption capacity, their protective effect is limited, which is aligned with the low inhibition observed in antityrosinase activity, indicating a partial effect on UV-induced damage and skin pigmentation processes.

**Table 3:** Assessment of spectrophotometric photoprotective potential.

Sample	Sun protective factor, SPF	Critical wavelength, λc (nm)	UVA/UVB ratio	Transmission of erythema	Transmission of pigmentation
*B. pilosa*	14.5 ± 0.2	352.0 ± 0.2	2.0 ± 0.0	1.3 ± 0.1	2.3 ± 0.1
*Croton* sp.	11.6 ± 0.4	350.7 ± 0.1	2.0 ± 0.0	2.3 ± 0.2	4.0 ± 0.4
Sunscreen	53.2 ± 0.2	391.0 ± 0.1	2.2 ± 0.0	0.0 ± 0.0	0.0 ± 0.0

Photoprotective potential of the extracts was evaluated at 500 µg/mL in absolute ethanol. Results reference a commercial broad-spectrum sunscreen using the laboratory’s established protocol. All values represent the mean ± standard deviation from triplicate measurements

#### Cytotoxic activity.

**Fig 1:**
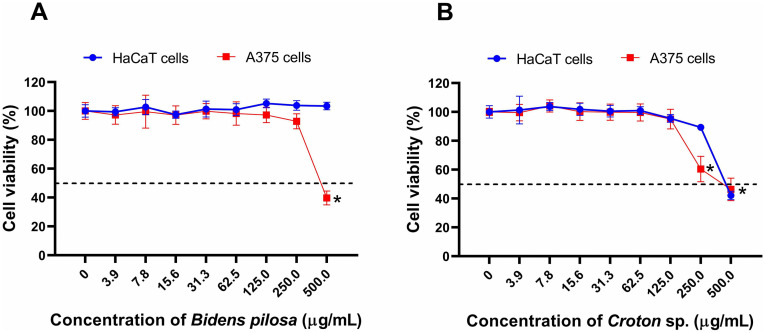
Viability of HaCaT keratinocytes and A375 melanoma cells exposed to the hydroalcoholic extracts of *B. pilosa* (A) and *Croton* sp. **(B)**. The asterisk (*) indicates significant difference in viability compared with the group of the non-treated cells (*p* < 0.05). Data are the mean ± SEM (n = 3).

The evaluation of the cytotoxicity of the hydroalcoholic extracts on HaCaT and A375 cells revealed distinct profiles (Fig 1, Table 4). *B. pilosa* did not show significant toxicity in HaCaT cells (IC₅₀ > 500 µg/mL), while in A375 melanoma cells it presented an IC₅₀ of 398.6 µg/mL, demonstrating the absence of antiproliferative effects on normal cells and suggesting a potential antimelanoma effect. In contrast, *Croton* sp. showed IC₅₀ of 329.5 µg/mL in HaCaT and 189.0 µg/mL in A375, with an SI of 1.74, indicating desirable selectivity towards tumor cells, but still below the recommended selectivity for a safe anticancer agent, suggesting the need for additional strategies, such as fractionation or formulation optimization, to improve its specificity.

**Table 4:** Determination of IC₅₀ and SI values in HaCaT keratinocytes and A375 human melanoma cell models.

Hydroalcoholic extract	IC₅₀ HaCaT (µg/mL)	IC₅₀ A375 (µg/mL)	SI (HaCaT/A375)	95% CI (HaCaT)	95% CI (A375)	R^2^ (HaCaT)	R^2^ (A375)
*B. pilosa*	>500	398.6	ND	ND	266.4 to 620.1	ND	0.5133
*Croton* sp.	329.5	189.0	1.74	156.5 to 693.7	125.1 to 285.6	0.3196	0.6570

50% Inhibitory Concentration (IC_50_). Selectivity index (SI). 95% Confidence Interval (95% CI). Not determined (ND)

## Discussion

The study of plant species from contrasting ecosystems is a priority in contemporary scientific research due to their potential as sources of bioactive compounds with applications in health and cosmetics [24–28]. These ecosystems, defined by heterogeneous environmental conditions that favor plant adaptation strategies, harbor a wide diversity of secondary metabolites of interest to different industrial sectors [29,30]. In this context, prospective studies are fundamental for advancing the identification, characterization, and sustainable use of these species. However, this process must recognize and value the traditional knowledge accumulated by local communities, where the ethnomedicinal use of plants has been preserved through generations as an essential part of their cultural identity [31,32]. Integrating this ethnocultural perspective with scientific research not only contributes to biodiversity conservation but also promotes respect for ancestral practices and fosters nature-based innovation [32]. Furthermore, the recovery and validation of this knowledge open opportunities for local economic development through the generation of value-added products, promoting productive diversification, improving quality of life, and fostering inclusive and sustainable bioeconomy models [33].

The findings of this study confirm that *B. pilosa* is a significant source of phenolic and flavonoid compounds, with a chemical profile dominated by rosmarinic acid and *p*-coumaric acid. The obtained total phenolic content (TPC, 21.1 mg GAE/g DT) and flavonoid content (TFC, 64.6 mg RE/g DT) evidence the antioxidant capacity of the extract, corroborated by FRAP assay (12,902.9 μmol TE/100 g) and TEAC-ABTS assay (25,183.4 μmol TE/100 g). These results are in line with previous reports, in which methanolic extracts of flowers and leaves presented TPC of 55.97–179.31 mg GAE/g and TFC of 29.33–165.63 mg QE/g (quercetin equivalent) [34–37]. Such variations reflect the influence of environmental factors (temperature, altitude, rainfall, soil composition), solvent used during extraction processes, and the plant organ on the concentration of bioactive metabolites [38,39].The enzymatic modulation exhibited by the hydroalcoholic extract of *B. pilosa*, characterized by moderate collagenase inhibition (53.9%), high hyaluronidase inhibition (64.5%), and low tyrosinase inhibition (11.1%), highlights its potential to protect the extracellular matrix and preserve hyaluronic acid, thereby contributing to the prevention of skin aging. This profile aligns with trends observed in species of the genus *Artemisia* (Asteraceae), which are widely recognized for their dermatological and cosmetic relevance [40]. Notably, *A. scoparia* Waldst. & Kit. and *A. princeps* Pamp. display collagenase inhibitions of 15.21% and 23.45%, tyrosinase inhibitions of 39.85% and 43.64%, and hyaluronidase inhibitions of 11.74% and 51.71%, respectively [41]. Although no previous reports describe the enzymatic activity of hydroalcoholic extracts of *B. pilosa*, studies using its supercritical extract have demonstrated significant improvements in collagen and elastin levels, reinforcing its potential as a promising agent for supporting extracellular matrix integrity [42]. The limited UV absorption (SPF 14.5; λc ≈ 351 nm; UVA/UVB ratio = 2.0) indicates that the photoprotective effects are more closely linked to antioxidant capacity than to direct radiation absorption, reaffirming that phenolics and flavonoids act as the main mediators of cellular protection [38]. In terms of cytotoxicity, the extracts were nontoxic to HaCaT keratinocytes (IC₅₀ > 500 μg/mL) and showed selective antimelanoma activity in A375 cells (IC₅₀ = 398.6 μg/mL), demonstrating a selectivity toward tumor cells. This is consistent with previous studies in which *B. pilosa* extracts inhibited the viability of KB-3–1, HeLa, and HepG2 with minimal toxicity to normal cells [19,43]. The identification of bioactive compounds such as paclitaxel, catechin, kaempferol, gallic acid, and ferulic acid by UHPLC-QqQLIT-MS/MS and GC-MS [43], supports the molecular basis of the observed antioxidant and cytotoxic effects. The results refine current models linking phenolic and flavonoid content to biological effects, highlighting the critical role of plant organs and extraction methods in metabolite yield. The evidence supports the premise that polar extracts optimize the recovery of phenolics and flavonoids with selective therapeutic effects [44–46]. Overall, *B. pilosa* is confirmed as a promising source of antioxidants, skin aging modulators, and especially as selective anticancer agents, highlighting its relevance in both therapeutic and cosmetic applications.

The hydroalcoholic extract of *Croton* sp. showed a high phenolic content (169.4 mg GAE/g DW) and flavonoid content (54.1 mg RE/g DW), values comparable to, and even higher than, those reported for other species of the genus. In *C. jacobinensis* Baill., for example, aqueous extracts reached 24,908.9 mg GAE/100 g, while the use of 70% ethanol optimized recovery to 32,521.5 mg GAE/100 g and an antioxidant capacity of 1,507.9 µM Trolox/g [47]. These data confirm that the combination of hydroalcoholic solvents maximizes the yield of bioactive metabolites [46]. The high antioxidant capacity observed in *Croton* sp. (FRAP: 14,999.1 µmol TE/100 g; TEAC-ABTS: 49,533.3 µmol TE/100 g) correlates with the presence of phenolic acids and flavonoids, consistent with values reported for *C. macrostachyus* Hochst. ex Delile (DPPH: 3.53–6.38 mg AAE/g) [48] and *C. grewioides* Baill*.*, where extracts showed DPPH inhibition above 80% and FRAP values up to 2,935.3 µmol Trolox/kg [49]. The variability between species and methods confirms that current models must consider both geographical origin and extraction technique as determinants of bioactivity. Regarding enzyme inhibition, *Croton* sp. showed a moderate effect on collagenase (55.0%), a high effect on hyaluronidase (76.5%), and a low effect on tyrosinase (12.7%), suggesting selectivity in enzyme modulation. These results open possibilities for dermocosmetic applications, although the limited UV absorption (SPF 11.6; λc ≈ 351 nm) restricts its potential as a primary sun protection agent. In terms of cytotoxicity, the extract showed an IC_50_ of 189.0 µg/mL in A375 cells and 329.5 µg/mL in HaCaT cells, with a selectivity index (SI) of 1.74, indicating moderate activity and a certain preference for tumor cells. This behavior is consistent with that of *C. lechleri* Müll.Arg., whose extracts showed IC_50_ values of 13.31–77.97 µg/mL in A375 cells and higher selectivity with Soxhlet ethanol (SI = 4.77) [27]. Similarly, diterpenes from *C. zambesicus* Müll.Arg. and *C. gratissimus* Burch. exhibited cytotoxicity in HeLa and HL-60 cell lines with IC_50_ values in the range of 7.3–32.6 µg/mL [50], confirming that specific metabolites can achieve significantly greater potency than crude extracts.

In conclusion, *B. pilosa* and *Croton* sp. emerge as promising botanical sources of bioactive compounds with relevance for antioxidant, anti-aging, and anticancer applications. The integration of phytochemical profiling, antioxidant evaluation, enzymatic modulation, and cytotoxicity assays demonstrates that both species possess substantial biological potential, although through distinct metabolite signatures and response patterns. Importantly, the findings show that the relationship between total phenolic and flavonoid content and biological activity cannot be attributed solely to quantitative abundance. Rather, bioactivity is driven by the specific chemical composition of each extract and by the potential synergistic interactions among metabolites, which collectively shape antioxidant performance, extracellular matrix protection, and selective cytotoxic effects. The results also underscore the critical influence of species identity, environmental conditions, plant organ, and extraction method on metabolite yield and functional activity. By refining and expanding current models linking phytochemical composition with biological effects, this study highlights the value of biodiversity and phytochemical approaches as strategic pathways for the development of natural ingredients with therapeutic and dermocosmetic potential.

Future studies should focus on identifying the specific metabolites responsible for the antioxidant, enzymatic, and cytotoxic effects observed in *B. pilosa* and *Croton* sp., as well as clarifying potential synergistic interactions between phenolic compounds and flavonoids. It will also be important to evaluate how environmental factors, plant organs, and extraction conditions influence bioactive profiles. Furthermore, the observed biological effects should be validated by using more advanced cell models and *in vivo* studies to confirm their safety and efficacy. Finally, standardization of the extracts should be ensured, and formulation strategies that improve stability and bioavailability should be explored.

## Materials and methods

### Plant material

Aerial parts of *B. pilosa* were collected at 3°00′57″ N, 76°30′51″ W (WGS84 datum) in an open, flat, and anthropogenically disturbed grassland in San Antonio village, Santander de Quilichao (Cauca Department, Colombia). Aerial parts of *Croton* sp. were collected in the vicinity of San Basilio de Palenque, Mahates municipality, Bolívar Department, Colombia (10°06′04.7″ N, 75°12′00.7″ W; WGS84 datum; approximately 100 m a.s.l.). The latter collection site is located in the lower foothills of the Montes de María, within a tropical savanna ecosystem characterized by open, moderately disturbed grasslands and a warm, seasonally dry climate. Plant collection permit was obtained from the Autoridad Nacional de Licencias Ambientales-ANLA (Colombia) under Resolution 001579 of July 25, 2024, and the Corporación Autónoma Regional del Canal del Dique-CARDIQUE (Colombia) under Resolution 0751 of June 27, 2014. Botanical identification was performed at the University of Atlantico (DUGAND Herbarium) and at the Colombian National Herbarium, respectively. The corresponding voucher specimens were deposited under the following accession numbers: *B. pilosa* (DUGAND 3525) and *Croton* sp. (COL 617816).

### Obtaining hydroalcoholic extracts

The dried and ground aerial parts of the plants (200 g) were extracted with 70% (v/v) ethanol under controlled conditions to favor the efficient extraction of polyphenols using eco-friendly solvents (ethanol, LiChrosolv, Merck, Darmstadt, Germany and Milli-Q^®^ water), using a sufficient amount to cover the plant material and maximize yield, in accordance with best practices carried out in our laboratory. After 24 h, the extracts were filtered through 125 mm filter paper (Albet^®^ LabScience, Dassel, Germany), and the plant material was subjected to hydroalcoholic extraction for an additional 24 h. The combined extracts were concentrated to 40 mL using a rotary evaporator (Scilogex RE100-Pro, Rocky Hill, Connecticut, USA) and subsequently freeze-dried (BK-FD12PT Biobase, Jinan, Shandong, China). The final products were stored at −20 °C in the dark. Solvent extractions were performed in duplicate. The extract yields were calculated as 6.3% for *B. pilosa* and 7.4% for *Croton* sp., respectively, using equation 1:


$$\text{Yield}\;(\%)=\;\frac{Weight\;of\;freeze-dried\;extract}{Weight\;of\;dry\;material\;used\;in\;maceration}*\text{100}$$ (1)

### Total phenolic content (TPC)

Total phenolic content was determined by the Folin-Ciocalteu method following the methodology of Sánchez-Gutiérrez et al. [51] with minor adaptations, using gallic acid as a standard (6–200 µg/mL) and plant extract (250 µg/mL). To each reaction mixture, 61.5 µL of sample or standard, 615.4 µL of Milli-Q^®^ water, 30.8 µL of 1 N Folin-Ciocalteu reagent, and 92.3 µL of 20% w/v sodium carbonate were added; the solutions were vortexed for 3 min. Subsequently, 200 µL of each preparation was dispensed into 96-well plates (three replicates per sample) and incubated for 2 h in the dark at room temperature. Absorbance was measured at 760 nm in a UV-Visible microplate reader (Varioskan LUX, Thermo), and total phenolic content was calculated from the gallic acid calibration curve, expressed as mg GAE/g TD. The data were statistically processed, obtaining the mean ± standard deviation.

Main characteristics of the methodology for the determination of total phenol contents are shown in Supplementary S2 Table.

### Total flavonoid content (TFC)

The total flavonoid content in the plant extracts was determined using the aluminum trichloride (AlCl₃) colorimetric method, based on the formation of complexes between aluminum ions and flavonoid hydroxyl groups, which produce chelates with characteristic absorbance between 385−440 nm, following the protocols previously reported [8,51,52]. For standardization, rutin was used as a reference, constructing a calibration curve (0.002–0.031 mg/mL). Reactions were performed with 2% AlCl₃, followed by vortexing and incubation at room temperature in the dark for 10 min. Absorbance was measured at 415 nm for extracts and standard. The results were expressed as mg of rutin equivalents per gram of dry tissue (mg RE/g TD), with triplicate replicates, and the results were expressed as the mean ± standard deviation. The main characteristics of the methodology for determining these biocompounds are summarized in Supplementary S3 Table.

### Chemical profile of extracts determined by UHPLC-ESI-Orbitrap-MS analysis

Chemical analysis of the extract was performed using a Dionex Ultimate 3000 ultra-high-performance liquid chromatography (UHPLC) system (Thermo Scientific), configured with a binary pump, autosampler, and thermostated column. Chromatographic separation was performed on a Hypersil GOLD Aq column (100 × 2.1 mm, 1.9 μm), using water and methanol as mobile phases, both modified with 0.1% formic acid and 5 mM ammonium formate. The elution program consisted of a linear gradient from 100% phase A to 100% phase B in 8 min, followed by a 4-min hold and 1-min re-equilibration, for a total run time of 13 min. Detection was performed using an Orbitrap high-resolution mass spectrometer, operated in positive electrospray ionization (ESI+) mode, with a capillary voltage of 3.5 kV. Data acquisition was performed in full-scan mode, with [M + H]^+^ ion current extraction, achieving a mass accuracy of <1 ppm and confirmation using isotopic and fragmentation standards. Compound quantification was carried out using calibration curves obtained with certified reference materials [9]. The main experimental conditions used in this analysis are summarized in Supplementary S4 Table.

### Antioxidant capacity

#### FRAP assay.

The FRAP assay relies on a single electron transfer (SET) reaction, where the ferric tripyridyltriazine complex [Fe³ ⁺ -(TPTZ)₂]³ ⁺ is reduced to its ferrous form [Fe² ⁺ -(TPTZ)₂]² ⁺ , producing an intense blue color under acidic conditions. To ensure reproducibility, the procedure was conducted as follows [9]: sample preparation (the extract stock solution was diluted 1:50 in type II water to maintain concentrations within the linear response range of the assay). Dispensing samples (using calibrated micropipettes, 10 μL of each diluted extract (or Trolox standard) were carefully transferred into separate wells of a 96-well microplate). Preparation of FRAP reagent (a fresh working solution was prepared by mixing three components in a 10:1:1 ratio: (a) 300 mM acetate buffer, pH 3.6; (b) 20 mM FeCl₃·6H₂O; and (c) 10 mM TPTZ dissolved in 40 mM HCl). Reaction setup (to each well containing the sample or standard, 250 μL of the FRAP reagent was added). Incubation (the plate was incubated in the dark at 37 °C for 10 min, allowing the redox reaction to proceed to completion). Measurement (absorbance was recorded at 593 nm using a BioTek Synergy HT multimode microplate reader BioTek Instruments, Inc., USA). Calibration and calculation (a Trolox calibration curve was generated with standard solutions covering the working concentration range. Absorbance values were converted into μmol TE/100 g). Validation and replication (linearity of the assay was verified (R² > 0.99). All samples and standards were measured in triplicate, and results were expressed as mean ± standard deviation).

#### ABTS assay.

The antioxidant capacity of the extracts was evaluated using the ABTS• ⁺ radical cation assay, adapted from the method of Bravo et al. [53], with minor modifications. Briefly, the crude extracts were dissolved in dimethyl sulfoxide to prepare a stock solution (100 mg/mL). Working solutions were obtained by diluting with methanol, for *B. pilosa* (1:50) and for *Croton* sp. (1:100), covering the expected linear range of the assay. The ABTS• ⁺ radical cation was generated by mixing ABTS (7 mM) with potassium persulfate (2.5 mM) in phosphate-buffered saline (PBS, pH 7.4). The mixture was left to stand at room temperature in the dark for 16 h to complete the oxidation. The oxidized ABTS• ⁺ solution was diluted with PBS to obtain an absorbance of 0.70 ± 0.02 at 730 nm. In each well of a 96-well microtiter plate, 20 µL of the diluted sample or Trolox standard was pipetted. Subsequently, 180 µL of the ABTS• ⁺ working solution was added. The plate was incubated at room temperature in the dark for 15 min to complete the reaction. Absorbance at 730 nm was measured using a Synergy HT microplate reader (BioTek Instruments, Inc., Winooski, USA). A calibration curve was prepared using Trolox standard solutions. Each measurement was performed in triplicate, and data are presented as mean ± standard deviation. The antioxidant capacity, expressed as Trolox Equivalent Antioxidant Capacity (TEAC) in µmol TE/100 g sample, was calculated based on the linear regression of the calibration curve, according to equation 2:


$$\frac{\mu mol\;TE}{\text{100}\;g\;sample}=\;\frac{\left(M*\;DF*\;V\right)}{m}*\text{100}$$ (2)

Where M is the molar concentration obtained from the calibration curve, DF the dilution factor applied to prepare the sample, V the volume in L in which the sample was initially prepared, and m the weight in g of the initial sample.

### Enzymatic inhibitory activity

The enzymatic inhibition of collagenase, hyaluronidase, and tyrosinase was evaluated using spectrophotometric assays that measured the absorbance of the samples compared to controls. The inhibition was quantified as a percentage, calculated using the model presented in equation 3:


$$Enzymatic\;Inhibition\;(\%)=\;\frac{M\;control-M\;sample}{M\;control}\;x\;\text{100}$$ (3)

Where, the variables M control and M sample correspond to the absorbance of control and sample, respectively.

The specific methodology applied for each enzyme is described below.

#### Collagenase inhibitory assay.

The collagenase inhibition assay was performed following the protocol of Barrantes and Guinea [54], with minor modifications. The collagenase enzyme from *Clostridium histolyticum* (Sigma-Aldrich) was prepared in 50 mM Tris-HCl buffer (pH 7.5, Sigma-Aldrich) supplemented with 10 mM calcium chloride (LOBA Chemie) and 400 mM sodium chloride (LOBA Chemie), resulting in a final concentration of 0.8 U/mL. This step ensured the presence of Ca² ⁺ , which is essential for enzyme activity. The crude extracts were dissolved in dimethyl sulfoxide to prepare a stock solution (100 mg/mL). The substrate FALGPA (N-[3-(2-Furyl)acryloyl]-Leu-Gly-Pro-Ala, Sigma-Aldrich) was dissolved in the same buffer at 2 mM, and for kinetic studies, serial dilutions between 0.5 and 2.5 mM were prepared. For the assay, 5 µL of the undiluted extract solution was diluted in 495 µL of buffer, while the negative control consisted of 5 µL of dimethyl in the same volume of buffer. In 96-well microplates, 25 µL of control or inhibitor, 25 µL of enzyme, and 50 µL of substrate were added to each well. Wells containing 25 µL of control or inhibitor and 75 µL of Tris-HCl buffer were included for absorbance correction. After a 15-min pre-incubation at 25 °C, the substrate was added, and the hydrolysis reaction was monitored by measuring absorbance at 340 nm immediately and every 2 min for 20 min using a Multiskan SkyHigh spectrophotometer (Thermo Scientific). The assay was performed in triplicate. EGCG (97%, Sigma-Aldrich) at 5 µM was used as a positive control. Results were expressed as mean ± standard deviation.

#### Hyaluronidase inhibitory assay.

The hyaluronidase inhibition assay was adapted from the method of Liyanaarachchi et al. [55] with slight modifications. The working solution of the extract was prepared by mixing 2.1 µL of the stock solution (crude extract in dimethyl sulfoxide at 100 mg/mL) with 18.9 µL of dimethyl sulfoxide in a 2 mL Eppendorf tube and then adding 179 µL of type I water. The control was prepared with 21 µL of dimethyl sulfoxide and 179 µL of water. For the enzyme mixture, 40 µL of bovine testicular hyaluronidase type 1-S (4200 U/mL in 0.1 M acetate buffer, pH 3.5; Sigma-Aldrich) was combined with 100 µL of the extract solution or control, and incubated for 20 min at 37 °C to allow enzyme-inhibitor interaction. Enzyme activation was performed by adding 40 µL of 12.5 mM calcium chloride (LOBA Chemie) and incubating at 37 °C for 10 min. Subsequently, 100 µL of sodium hyaluronate (12 mg/mL in 0.1 M acetate buffer, pH 3.5; USP) was added, and incubated for 40 min at 37 °C for substrate hydrolysis. The reaction was stopped by adding 20 µL of 0.9 M NaOH and 40 µL of 0.2 M sodium borate, followed by incubation in a boiling water bath for 3 min. After cooling, 100 µL of p-dimethylaminobenzaldehyde (LOBA Chemie) was added, and incubated for 10 min at 37 °C to develop color. Finally, 150 µL of each sample were transferred in duplicate to 96-well microplates, and the absorbance was measured at 585 nm using a Multiskan SkyHigh spectrophotometer (Thermo Scientific). EGCG (97%, Sigma-Aldrich) at 10 µM was used as a positive control. Data were expressed as mean ± standard deviation.

#### Tyrosinase inhibitory assay.

The depigmenting potential of the extracts was evaluated using a tyrosinase inhibition assay, previously reported [9]. Enzymatic activity was measured from the oxidation of L-tyrosine to L-DOPA and subsequently to L-dopaquinone, the formation of which was recorded spectrophotometrically at 480 nm. For the assay, each sample was prepared at a final concentration of 0.25 mg/mL in phosphate buffer (50 mM, pH 6.5, Sigma-Aldrich). Kojic acid (0.100 mM, Sigma-Aldrich) was used as a positive control. In each well of a 96-well microplate (Corning Inc.), 70 µL of the sample solution was added, followed by 30 µL of mushroom tyrosinase (333 U/mL, Sigma-Aldrich). After 5 min of incubation at room temperature, the reaction started with 110 µL of L-tyrosine (2 mM, Sigma-Aldrich). The absorbance was recorded every minute for 20 min at 480 nm using a BioTek Synergy HT multimode reader (BioTek Instruments, Inc., Winooski, VT, USA). The percentage of inhibition was determined by comparing the signal obtained with the buffer control, and the results were expressed as the mean ± standard deviation of three independent replicates.

### Spectrophotometric UV-protective potential

The UV protection potential of the extracts was evaluated by spectrophotometric analysis using BioTek Synergy HT multimode microplate reader BioTek Instruments, Inc., USA. Absorption spectrum was recorded between 290 and 400 nm. Photoprotection was assessed using five complementary indices: SPF (calculated from absorbance values in the UVB region (290–320 nm) using the Mansur equation, applying a correction factor, CF = 10); λc (determined from the area under the absorption curve between 290 and 400 nm, identifying the wavelength at which 90% of the cumulative absorbance is reached. Products with λc ≥ 370 nm were considered broad spectrum according to FDA guidelines); UVA/UVB ratio (calculated as the ratio of mean absorbance between the UVA (320–400 nm) and UVB (290–320 nm) regions, interpreted according to the Boots star rating system); erythema transmission (estimated from absorbance data in the 292–338 nm range, reflecting the samples’ ability to reduce erythematous radiation); and pigmentation transmission (calculated from absorbance between 322 and 372 nm, indicating protection against pigmentation-inducing radiation). All measurements were performed in triplicate at a concentration of 500 µg/mL, employing absolute ethanol as solvent and blank. Photoprotection efficacy was classified according to international standards and previously established protocols [1,8,10,28,56–60].

The calculation models, evaluation criteria, and constant values used in the analyses are presented in Supplementary S5-S8 Tables.

### Cytotoxicity on keratinocytes and human melanoma cell lines

Cytotoxicity was assessed in HaCaT keratinocytes (cat. no. 300493, CLS Cell Lines Service GmbH, Germany) and A375 melanoma cells (cat. no. 300110, CLS Cell Lines Service LLC, USA) using the colorimetric MTT assay, adapted with minor modifications from Caballero-Gallardo et al. [8]. Cells were grown in Dulbecco’s modified Eagle’s medium (DMEM, Sigma-Aldrich) supplemented with 10% fetal bovine serum and 1% penicillin/streptomycin and seeded in 96-well plates at a density of 1.5 × 10⁴ cells per well. Cultures were incubated at 37 °C in a humidified atmosphere with 5% CO₂ until approximately 80% confluence was reached. Each extract was prepared from a 100 mg/mL stock solution in dimethyl sulfoxide. Serial 1:2 dilutions of the extract were made in DMEM to obtain final concentrations ranging from 3.9 to 500.0 μg/mL. After 24 h of exposure, cells were washed with phosphate-buffered saline (PBS, Sigma-Aldrich), and 50 μL of MTT reagent (thiazolyl blue tetrazolium bromide, 5 mg/mL; Millipore) was added to each well. After a 3-h incubation, the medium was removed, and the formazan crystals were solubilized in 200 μL of dimethyl sulfoxide. Absorbance was measured at 570 nm using a multimode microplate reader (Varioskan™ LUX, Thermo Fisher Scientific, Inc.), and cell viability was expressed as a percentage relative to untreated controls using the following equation:


$$Cell\;viability\;(\%)=\;\frac{Absorbance\;of\;the\;test\;\;}{Absorbance\;of\;control\;}\;x\;100$$ (4)

Finally, the selectivity index (SI) was calculated as the ratio between IC₅₀ in normal cells and IC₅₀ in target cells, where SI > 1 indicates selectivity for tumor or target cells, and SI > 3 reflects high selectivity [61,62].

### Statistical analysis

The data obtained were analyzed using descriptive statistical techniques, expressing the results as the mean ± standard deviation. In cytotoxicity assays, data normality was assessed using the Shapiro-Wilk test. To compare differences between groups, a one-way ANOVA was performed, followed by Dunnett’s post-hoc test to identify significant differences compared to the control. IC₅₀ values were determined by nonlinear regression using a four-parameter logistic model with 95% confidence intervals. Experiments were performed in quadruplicate and repeated twice independently, and statistical significance was set at *p* < 0.05.

## Supporting information

S1 Table Identification and quantification of 20 phytocompounds by UHPLC-ESI-Orbitrap-HRMS. (PDF)

S2 Table Main characteristics of the methodology for the determination of total phenolic contents. (PDF)

S3 Table Main characteristics of the methodology for the determination of total flavonoid contents. (PDF)

S4 Table Experimental conditions for chemical analysis by UHPLC-ESI-Orbitrap-MS. (PDF)

S5 Table Evaluation criteria of photoprotection indices. (PDF)

S6 Table Photoprotection indices calculation model. (PDF)

S7 Table EE(λ) x I(λ) constant values to wavelength determinate. (PDF)

S8 Table Erythema and pigmentation flux constant values on sunscreens to wavelength determinate. (PDF)

S1 File Minimal Data set. (ZIP)
