# Supplementary material for: Phytochemical characterization, total phenolic and flavonoid content, antioxidant capacity, enzymatic profiling, and cytotoxicity of Bidens pilosa and Croton sp. from Colombia for applications in skin health
Source: PLoS One. 2026 Jan 9;21(1):e0340869. doi: 10.1371/journal.pone.0340869 (PMC12788638; doi:10.1371/journal.pone.0340869)
Supplement: S1 Table — (PDF) [file pone.0340869.s001.pdf]

Table S1. Identification and quantification of 20 phytocompounds by UHPLC-ESI-Orbitrap-HRMS

No.	Compound	T_R [a], min	MQL [b], mg/kg	***Bidens pilosa***	***Crotons sp.***
				**Concentration expressed in mg/kg of dry sample**	
1	Theobromine	2.9	0.4	<0.4	<0.4
2	Theophylline	3.4	0.4	<0.4	<0.4
3	*p*-Hydroxybenzoic acid	3.4	0.4	<0.4	<0.4
4	Caffeine	3.8	0.4	<0.4	<0.4
5	Caffeic acid	3.9	0.4	<0.4	<0.4
6	Epigallocatechin gallate	3.9	0.4	<0.4	<0.4
7	Epicatechin	4.0	0.4	<0.4	<0.4
8	Vanillic acid	4.3	0.4	<0.4	<0.4
9	*p*-Coumaric acid	4.5	0.4	**0.7**	**2.9**
10	Epicatechin gallate	4.5	0.4	<0.4	<0.4
11	Ferulic acid	5.2	0.4	<0.4	<0.4
12	Rosmarinic acid	5.2	10.0	**67.2**	**55.9**
13	Rutin	5.2	0.4	<0.4	<0.4
14	*trans*-Cinnamic acid	5.9	2.0	<2.0	<2.0
15	Quercetin	6.0	0.4	<0.4	**20.4**
16	Naringenin	6.0	0.4	<0.4	<0.4
17	Luteolin	6.2	0.4	<0.4	<0.4
18	Apigenin	6.5	0.4	<0.4	<0.4
19	Pinocembrin	6.9	0.4	<0.4	<0.4
20	Ursolic acid	9.3	0.4	<0.4	<0.4

[a] Retention time (T_R). [b] Minimum quantification level (MQL).
