# Supplementary material for: Phytochemical characterization, total phenolic and flavonoid content, antioxidant capacity, enzymatic profiling, and cytotoxicity of Bidens pilosa and Croton sp. from Colombia for applications in skin health
Source: PLoS One. 2026 Jan 9;21(1):e0340869. doi: 10.1371/journal.pone.0340869 (PMC12788638; doi:10.1371/journal.pone.0340869)
Supplement: S2 Table — (PDF) [file pone.0340869.s002.pdf]

Table S2. Main characteristics of the methodology for the determination of total phenolic contents

Stage	Condition / Equipment used
Method[a]	Folin–Ciocalteu method
Main reagents	2 N Folin–Ciocalteu reagent (Sigma-Aldrich), Milli-Q® water, Na_2CO_3 (Sigma-Aldrich), gallic acid (Sigma-Aldrich)
Construction of the standard curve	Gallic acid in Milli-Q® water (6.25–200 µg/mL)
Extract concentration	250 µg/mL in Milli-Q® water
Reaction mixture	Extract/standard (61.5 µL) + Milli-Q® water (615.4 µL) + 1 N Folin–Ciocalteu (30.8 µL) + 20% w/v Na_2CO_3 (92.3 µL); Final volume: 800 µL
Stirring	Vortex mixing, 3 min
Dispensed	200 µL into 96-well plates (3 replicates)
Incubation	2 h, dark, room temperature
Absorbance reading	760 nm in a UV/VIS microplate reader (Varioskan LUX, Thermo)
Calibration curve	$A_{760\ nm} = 0.0049x – 0.0134$ ($R^2 = 0.9957$); range: 6.25–200 µg/mL
Expression of results	mg gallic acid equivalents per g of dry tissue (mg GAE/g DT)
Statistical analysis	Mean ± standard deviation

[a] The determination was performed according to the protocol described by Sánchez-Gutiérrez et al. 2019, applied with minor adjustments.

Reference

Sánchez-Gutiérrez, J. A., Moreno-Lorenzana, D., Álvarez-Bernal, D., Rodríguez-Campos, J. & Medina-Medrano, J. R. (2019). Phenolic profile, antioxidant and anti-proliferative activities of methanolic extracts from *Asclepias linaria* cav. Leaves. Molecules, 25 (1), 54.
