# Supplementary material for: Phytochemical characterization, total phenolic and flavonoid content, antioxidant capacity, enzymatic profiling, and cytotoxicity of Bidens pilosa and Croton sp. from Colombia for applications in skin health
Source: PLoS One. 2026 Jan 9;21(1):e0340869. doi: 10.1371/journal.pone.0340869 (PMC12788638; doi:10.1371/journal.pone.0340869)
Supplement: S3 Table — (PDF) [file pone.0340869.s003.pdf]

Table S3. Main characteristics of the methodology for the determination of total flavonoid contents

Stage	Condition / Equipment used
Method[a]	Colorimetric method with $AlCl_3 \cdot 6H_2O$ (flavonoid-Al(III) chelate)
Main reagents	$AlCl_3 \cdot 6H_2O$ (Sigma-Aldrich), methanol (Sigma-Aldrich), Milli-Q® water, standard rutin (Sigma-Aldrich), plant extract
Construction of the standard curve	Rutin (Sigma-Aldrich), curve 2–31.5 µg/mL
Extract concentration	250 µg/mL in methanol
Reaction mixture	400 µL rutin/extract + 400 µL $AlCl_3$ 2% (standard/extract) or + 400 µL Milli-Q® water (control); Final volume: 800 µL
Stirring	Vortex mixing, 3 min
Dispensed	200 µL into 96-well plates (3 replicates)
Incubation	10 min, in the dark, at room temperature
Absorbance reading	415 nm (chelate rutin-$AlCl_3$) in a UV/VIS microplate reader (Varioskan LUX, Thermo)
Calibration curve	$A_{415\ nm} = 0.0095\ x - 0.0014$ ($R^2 = 1$); Range: 2–31.5 µg/mL.
Expression of results	mg rutin equivalents per g of dry tissue (mg RE/g DT)
Statistical analysis	Mean ± standard deviation

[a] The determination was performed according to the protocol described by Sánchez-Gutiérrez et al. 2019, applied with minor adjustments.

Reference

Sánchez-Gutiérrez, J. A., Moreno-Lorenzana, D., Álvarez-Bernal, D., Rodríguez-Campos, J. & Medina-Medrano, J. R. (2019). Phenolic profile, antioxidant and anti-proliferative activities of methanolic extracts from *Asclepias linaria* cav. Leaves. Molecules, 25 (1), 54.
