# Supplementary material for: Phytochemical characterization, total phenolic and flavonoid content, antioxidant capacity, enzymatic profiling, and cytotoxicity of Bidens pilosa and Croton sp. from Colombia for applications in skin health
Source: PLoS One. 2026 Jan 9;21(1):e0340869. doi: 10.1371/journal.pone.0340869 (PMC12788638; doi:10.1371/journal.pone.0340869)
Supplement: S4 Table — (PDF) [file pone.0340869.s004.pdf]

Table S4. Experimental conditions for chemical analysis by UHPLC-ESI-Orbitrap-MS

Stage	Condition / Equipment used
Sample preparation	MeOH:H_2O (1:1, v/v) + 0.2% HCOOH; vortex 5 min; sonication 20 min
UHPLC System	Dionex Ultimate 3000 (Thermo Scientific), binary pump, autosampler, thermostatted column
Chromatographic column	Hypersil GOLD Aq (100 × 2.1 mm, 1.9 µm)
Mobile phases	A: Water + 0.1% HCOOH + 5 mM ammonium formate<br>B: MeOH + 0.1% HCOOH + 5 mM ammonium formate
Elution Program	Gradient: 100% A → 100% B in 8 min; hold for 4 min; re-equilibrate for 1 min (total 13 min)
Detection (MS)	Orbitrap HRMS, ESI+ mode, capillary voltage 3.5 kV
Data acquisition	Full-scan; [M+H]+ extraction; Δppm < 1; isotopic and fragmentation review
Quantification	Calibration curves with certified standards:<br>theobromine (Sigma-Aldrich, 99%), theophylline (Sigma-Aldrich, 99%), *p*-hydroxybenzoic acid (Sigma-Aldrich, 99.9%), caffeine (Sigma-Aldrich, 99.9%), Caffeic acid (Sigma-Aldrich, 99.5%), (-)-epigallocatechin gallate (EGCG) (PhytoLab, 99.5%), (-)-epicatechin (EC) (Sigma-Aldrich, 95.1%), vanillic acid (STD-Aldrich, 97%), *p*-coumaric acid (Sigma-Aldrich, 98.6%), (-)-epicatechin gallate (ECG) (PhytoLab, 98.5%), ferulic acid (Sigma-Aldrich, 99.6%), rosmarinic acid (Sigma-Aldrich, 97%), *trans*-cinnamic acid (Sigma-Aldrich, 99%), rutin (Sigma-Aldrich, 97%), quercetin (Sigma-Aldrich, 98%), naringenin (Sigma-Aldrich, 98.6%), luteolin (Sigma-Aldrich, 98%), apigenin (AK Scientific LCMS, 99.2%), pinocembrin (Sigma-Aldrich, 95%), and ursolic acid (Sigma-Aldrich, 99.6%).
