# Supplementary material for: Phytochemical characterization, total phenolic and flavonoid content, antioxidant capacity, enzymatic profiling, and cytotoxicity of Bidens pilosa and Croton sp. from Colombia for applications in skin health
Source: PLoS One. 2026 Jan 9;21(1):e0340869. doi: 10.1371/journal.pone.0340869 (PMC12788638; doi:10.1371/journal.pone.0340869)
Supplement: S5 Table — (PDF) [file pone.0340869.s005.pdf]

**Table S5.** Evaluation criteria of photoprotection indices

| Index | Spectral range (nm) | Calculation principle | Interpretation criteria | Reference |
|---|---|---|---|---|
| Sun protective factor (SPF) | 290–320 | Mansur equation with CF = 10 | SPF numerical value: SPF 2-15 (low protection) SPF 15-30 (moderate protection) SPF 30-50 (high protection) SPF >50 (maximum protection) | Mansur et al. 1986; Caballero-Gallardo et al. 2022 |
| Critical wavelength (λc) | 290–400 | 90% of cumulative absorbance | λc ≥ 370 nm = broad spectrum | Springsteen et al. 1999; Caballero-Gallardo et al. 2022 |
| UVA/UVB ratio | 290–400 | Mean UVA / mean UVB absorbance | Boots star system (* to ****): The star rating system indicates that 0.0 to <0.2 is too low for UVA protection (-), 0.2 to <0.4 is a moderate protector (*), 0.4 to <0.6 is a good protector (**), 0.6 to <0.8 is a superior protector (***), | Panyakaew et al. 2021; Caballero-Gallardo et al. 2022 |

| | | | | |
|---|---|---|---|---|
| | | | and 0.8 to ≥0.8 is a maximum protector (****) | |
| Transmission of erythema (%) | 292–338 | From transmittance and Fe constants | Categories:<br>Sunscreen, <1<br>Extra protection, 1-6<br>Standard tan, 6-12<br>Quick tan, 10-18 | Sami et al. 2021; Caballero-Gallardo et al. 2022 |
| Transmission of pigmentation (%) | 322–372 | From transmittance and Fp constants | Categories:<br>Sunscreen, 3-40<br>Extra protection, 42-86<br>Standard tan, 45-86<br>Quick tan, 45-86 | Sami et al. 2021; Caballero-Gallardo et al. 2022 |

## Reference


Caballero-Gallardo K, Quintero-Rincón P, Stashenko EE, Olivero-Verbel J. Photoprotective agents obtained from aromatic plants grown in Colombia: total phenolic content, antioxidant activity, and assessment of cytotoxic potential in cancer cell lines of *Cymbopogon flexuosus* L. and *Tagetes lucida* Cav. essential oils. Plants. 2022;11:1693.

DeSouzaMansur J, Breder MNR, d'Ascensão Mansur MC, Azulay RD. Determination of sun protecting factor in human beings and by spectrophotometry: comparison between the two methods. An Bras Dermatol. 1986;61:167-72.

Panyakaew J, Chalom S, Sookkhee S, Saiai A, Chandet N, Meepowpan P, et al. *Kaempferia* sp. extracts as UV protecting and antioxidant agents in sunscreen. J Herbs Spices Med Plants. 2021;27:37-56.

Radice M, Manfredini S, Ziosi P, Dissette V, Buso P, Fallacara A, et al. Herbal extracts, lichens and biomolecules as natural photo-protection alternatives to synthetic UV filters: a systematic review. Fitoterapia. 2016;114:144-62.

Sami FJ, Soekamto NH, Latip J. Bioactivity profile of three seaweeds as antioxidant, UV-protection as sunscreen and their correlation activity. Food Res. 2021;5:441-7.

Springsteen A, Yurek R, Frazier M, Carr KF. *In vitro* measurement of sun protection factor of sunscreens by diffuse transmittance. Anal Chim Acta. 1999;380:155-64.
