# Supplementary material for: Phytochemical characterization, total phenolic and flavonoid content, antioxidant capacity, enzymatic profiling, and cytotoxicity of Bidens pilosa and Croton sp. from Colombia for applications in skin health
Source: PLoS One. 2026 Jan 9;21(1):e0340869. doi: 10.1371/journal.pone.0340869 (PMC12788638; doi:10.1371/journal.pone.0340869)
Supplement: S6 Table — (PDF) [file pone.0340869.s006.pdf]

Table S6. Photoprotection indices calculation model

Evaluated parameter	General considerations
Sun Protective Factor (SPF)	Absorbance is measured in the wavelength range 290 to 320 nm (UVB), using an optical path of 5.0 cm. To obtain the SPF values, the Mansur equation should be applied: $$\text{SPF} = \text{CF x} \sum_{290\ \text{nm}}^{320\ \text{nm}} \text{EE}\ (\lambda) \text{x}\ \text{I}(\lambda) \text{x Abs}\ (\lambda)$$
Critical wavelength (λc)	Absorbance is measured in the wavelength range of 290 to 400 nm (UVA and UVB), using an optical path of 1.0 cm. To determine λc values, the following equation is applied: $$\int_{290\ \text{nm}}^{\lambda c} \text{A}\ (\lambda) d\lambda = 0.9 \int_{290\ \text{nm}}^{400\ \text{nm}} \text{A}(\lambda) d\lambda$$
UVA/UVB ratio	Absorbance is measured in the wavelength range of 290 to 400 nm (UVA and UVB), using an optical path of 5.0 cm. To determine the UVA/UVB ratio, the following equation is applied: $$\frac{\text{UVA}}{\text{UVB}} = \frac{\sum_{320\ \text{nm}}^{400\ \text{nm}} \text{A}(\lambda), \text{d}(\lambda)}{\sum_{290\ \text{nm}}^{320\ \text{nm}} \text{A}(\lambda), \text{d}(\lambda)}$$
Transmission of erythema (%)	Absorbance is measured in the wavelength range of 292 to 372 nm (UVA and UVB), using an optical path of 5.0 cm. It is necessary calculates transmission (T) using A = 1/ T = - log T. Then, the following equation is applied: $$\text{Transmission of erythema}\ (\%) = \frac{\text{Ee}}{\sum \text{Fe}} = \sum \frac{(\text{T x Fe})}{\sum \text{Fe}}$$
Transmission of pigmentation (%)	Absorbance is measured in the wavelength range of 292 to 372 nm (UVA and UVB), using an optical path of 5.0 cm. It is necessary calculates transmission (T) using A = 1/ T = - log T. Then, the following equation is applied: $$\text{Transmission of pigmentation}\ (\%) = \frac{\text{Ep}}{\sum \text{Fp}} = \sum \frac{(\text{T x Fp})}{\sum \text{Fp}}$$

Taken and modified from Quintero-Rincón et al. 2023

Reference

Quintero-Rincón P, Pino-Benítez N, Galeano E, Rojo-Uribe C, Mesa-Arango AC, Flórez-Acosta OA. *Sloanea chocoana* and *S. pittieriana* (Elaeocarpaceae): Chemical and Biological Studies of Ethanolic Extracts and Skincare Properties. Plants. 2023;12(23):3953.
