# Supplementary material for: Phytochemical characterization, total phenolic and flavonoid content, antioxidant capacity, enzymatic profiling, and cytotoxicity of Bidens pilosa and Croton sp. from Colombia for applications in skin health
Source: PLoS One. 2026 Jan 9;21(1):e0340869. doi: 10.1371/journal.pone.0340869 (PMC12788638; doi:10.1371/journal.pone.0340869)
Supplement: S7 Table — (PDF) [file pone.0340869.s007.pdf]

Table S7. EE(λ) x I(λ) constant values to wavelength determinate

**Wavelength (λ, nm)**	**EE(λ) x I(λ)**
290	0.015
295	0.082
300	0.287
305	0.328
310	0.186
315	0.084
320	0.018

Taken from Caballero-Gallardo et al. 2022

Reference

Caballero-Gallardo K, Quintero-Rincón P, Stashenko EE, Olivero-Verbel J. Photoprotective agents obtained from aromatic plants grown in Colombia: total phenolic content, antioxidant activity, and assessment of cytotoxic potential in cancer cell lines of *Cymbopogon flexuosus* L. and *Tagetes lucida* Cav. essential oils. Plants. 2022;11:1693.
