# Supplementary material for: Phytochemical characterization, total phenolic and flavonoid content, antioxidant capacity, enzymatic profiling, and cytotoxicity of Bidens pilosa and Croton sp. from Colombia for applications in skin health
Source: PLoS One. 2026 Jan 9;21(1):e0340869. doi: 10.1371/journal.pone.0340869 (PMC12788638; doi:10.1371/journal.pone.0340869)
Supplement: S8 Table — (PDF) [file pone.0340869.s008.pdf]

Table S8. Erythema and pigmentation flux constant values on sunscreens to wavelength determinate

Wavelength (λ; nm)	Flux of erythema (Fe)	Flux of pigmentation (Fp)
290-295	11.390	
295-300	65.100	
300-305	100.000	
305-310	35.770	
310-315	0.973	
315-320	0.567	
320-325	337.500	10.790
325-330	0.289	10.200
330-335	0.129	0.936
335-340	0.046	0.798
340-345		0.669
345-350		0.570
350-355		0.488
355-360		0.456
360-365		0.356
365-370		0.310
370-375		0.260
Σ	551.764	25.833

Taken from Caballero-Gallardo et al. 2022

Reference

Caballero-Gallardo K, Quintero-Rincón P, Stashenko EE, Olivero-Verbel J. Photoprotective agents obtained from aromatic plants grown in Colombia: total phenolic content, antioxidant activity, and assessment of cytotoxic potential in cancer cell lines of *Cymbopogon flexuosus* L. and *Tagetes lucida* Cav. essential oils. Plants. 2022;11:1693.
