# Supplementary material for: Phytochemical characterization, total phenolic and flavonoid content, antioxidant capacity, enzymatic profiling, and cytotoxicity of Bidens pilosa and Croton sp. from Colombia for applications in skin health
Source: PLoS One. 2026 Jan 9;21(1):e0340869. doi: 10.1371/journal.pone.0340869 (PMC12788638; doi:10.1371/journal.pone.0340869)
Supplement: S1 File — (ZIP) [file pone.0340869.s009.zip › Minimal Data Set/Binder1.pdf]

C:\Xcalibur\...\241010AG\994708-01-EB

RT: 0.00 - 13.01 SM: 9G

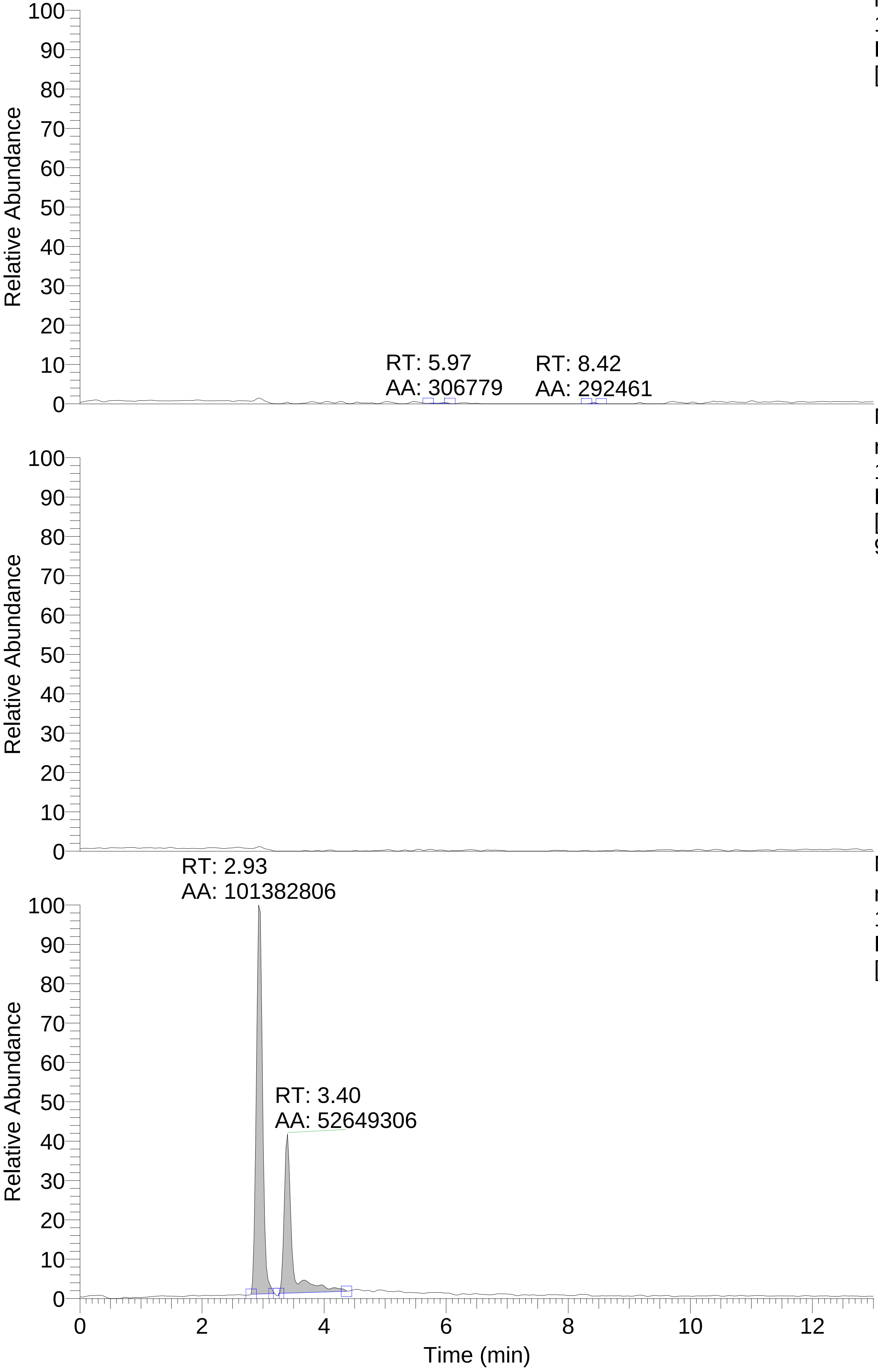

NL: 1.44E7
m/z=
181.07109-181.07291 F:
FTMS + p ESI Full ms
[80.0000-1000.0000] MS
Genesis 994708-01-EB

NL: 1.44E7
m/z=
181.07109-181.07291 F:
FTMS + p ESI Full ms
[80.0000-1000.0000] MS
994708-02-eb

NL: 1.44E7
m/z=
181.07109-181.07291 F:
FTMS + p ESI Full ms
[80.0000-1000.0000] MS
Genesis polifenoles-p6

c:\xcalibur\...\994708-02-eb-d200

RT: 0.00 - 13.01 SM: 9G

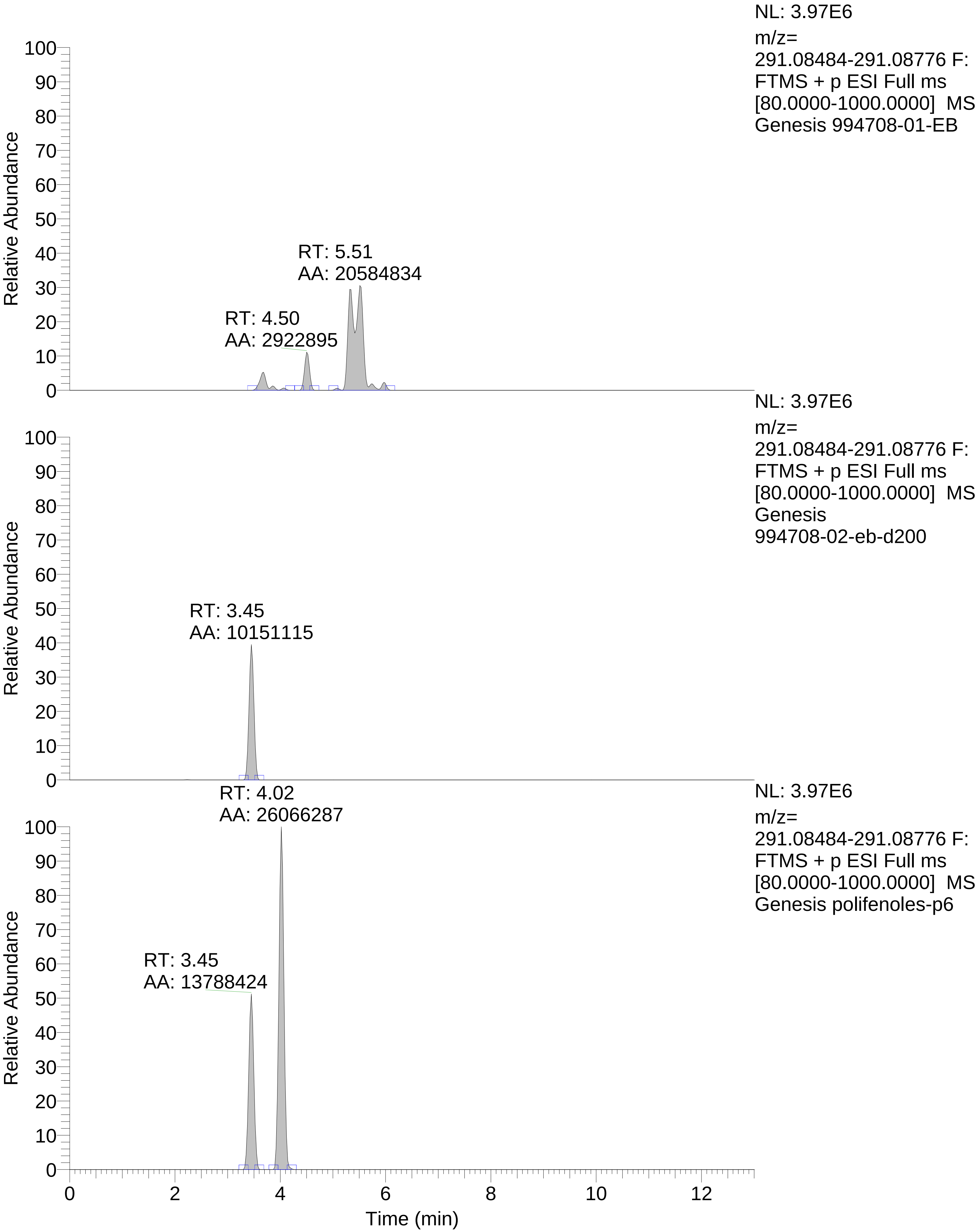

C:\Xcalibur\...\241010AG\994708-01-EB

RT: 0.00 - 13.01 SM: 9G

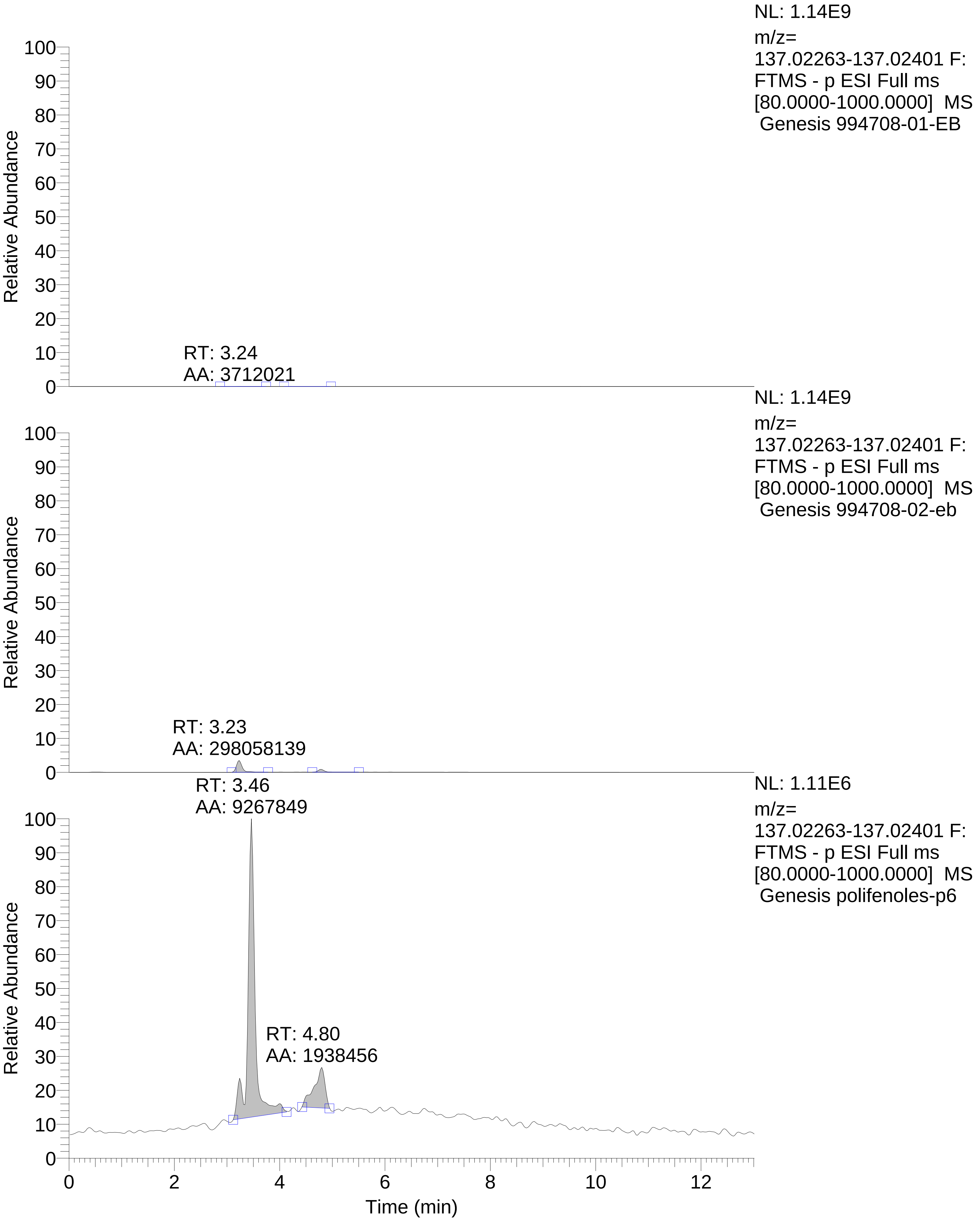

C:\Xcalibur\...\241010AG\994708-01-EB 10/11/24 01:40:20

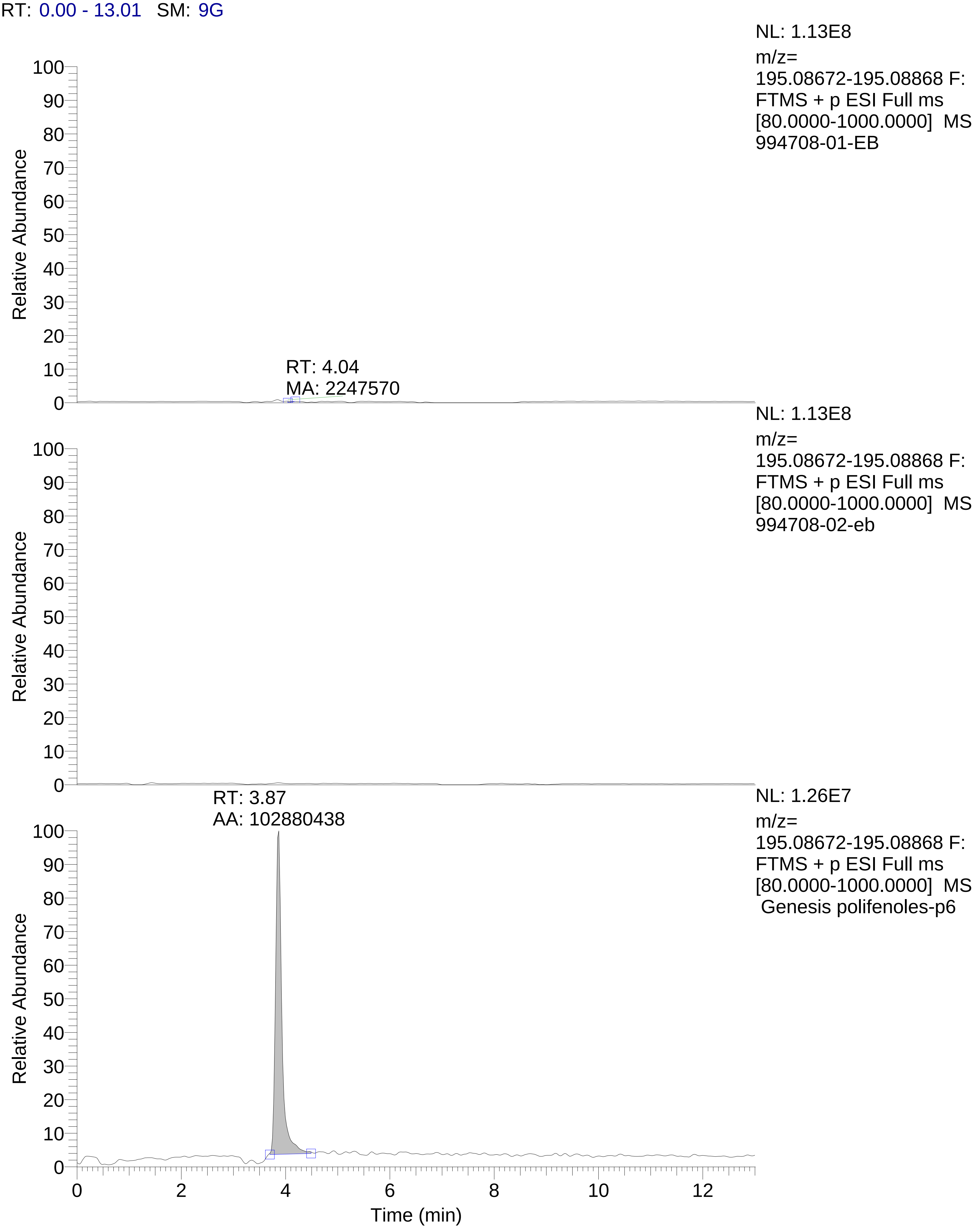

RT: 0.00 - 13.01 SM: 9G

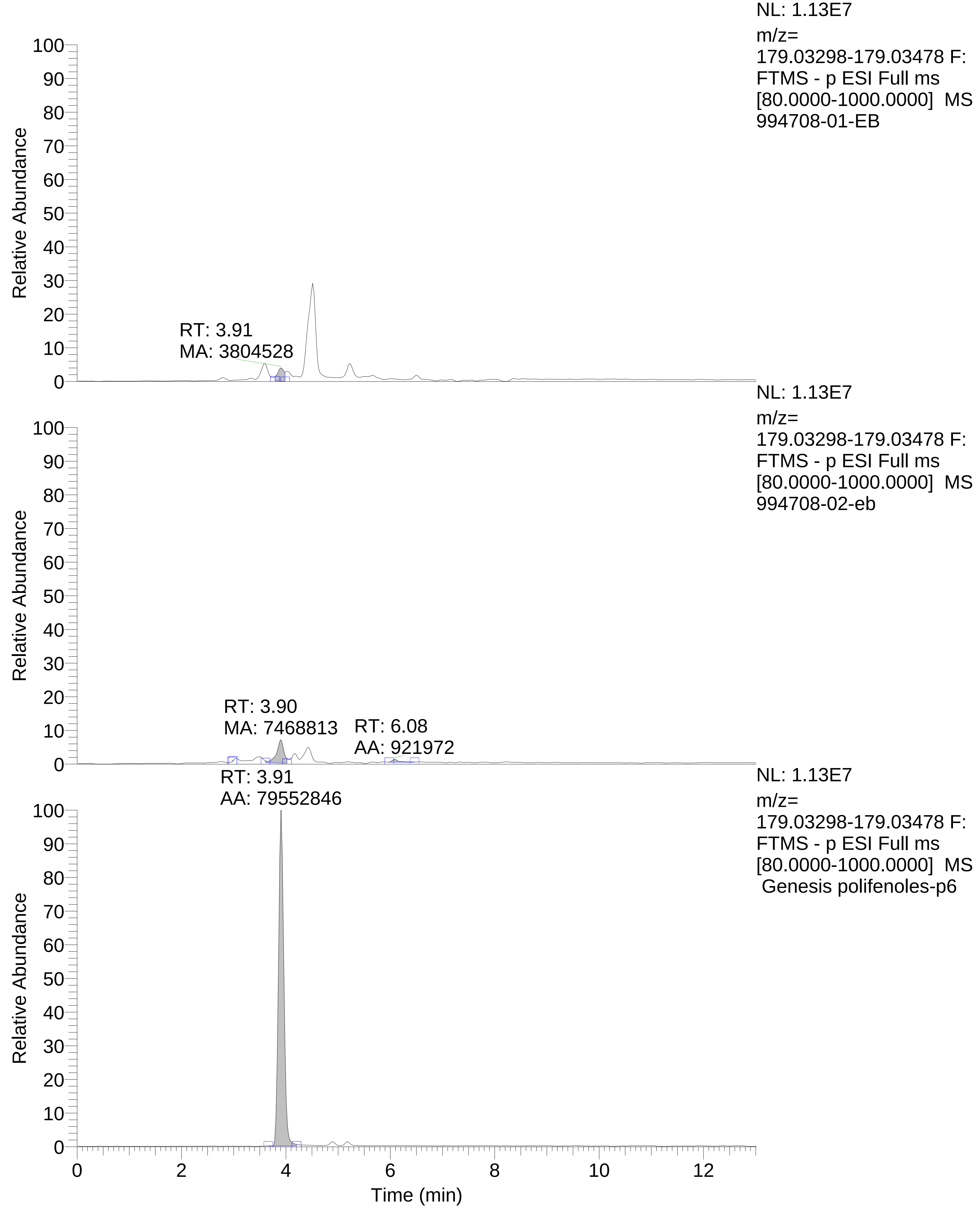

C:\Xcalibur\...\241010AG\994708-01-EB

RT: 0.00 - 13.01 SM: 9G

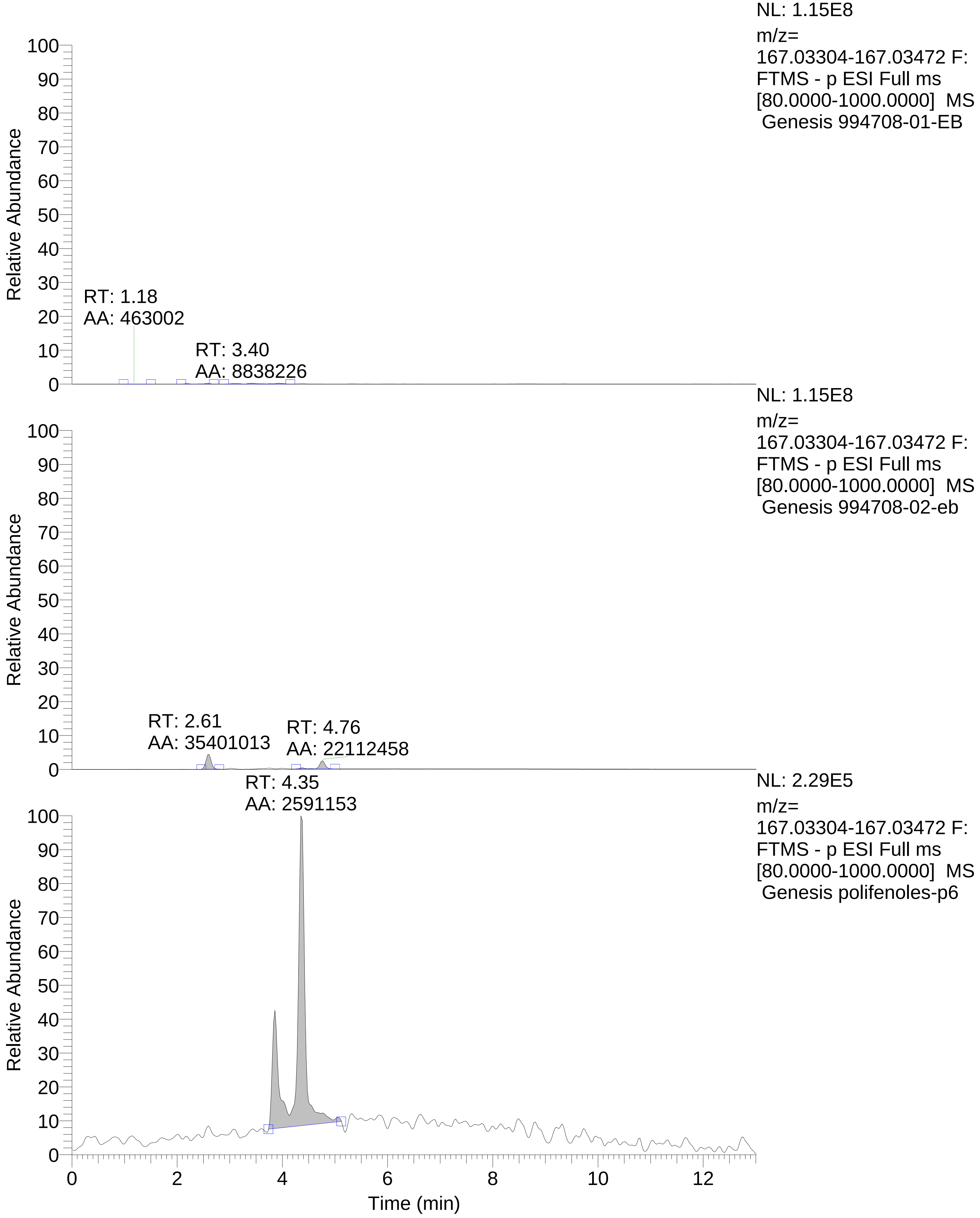

C:\Xcalibur\...\241010AG\994708-01-EB

RT: 0.00 - 13.01 SM: 9G

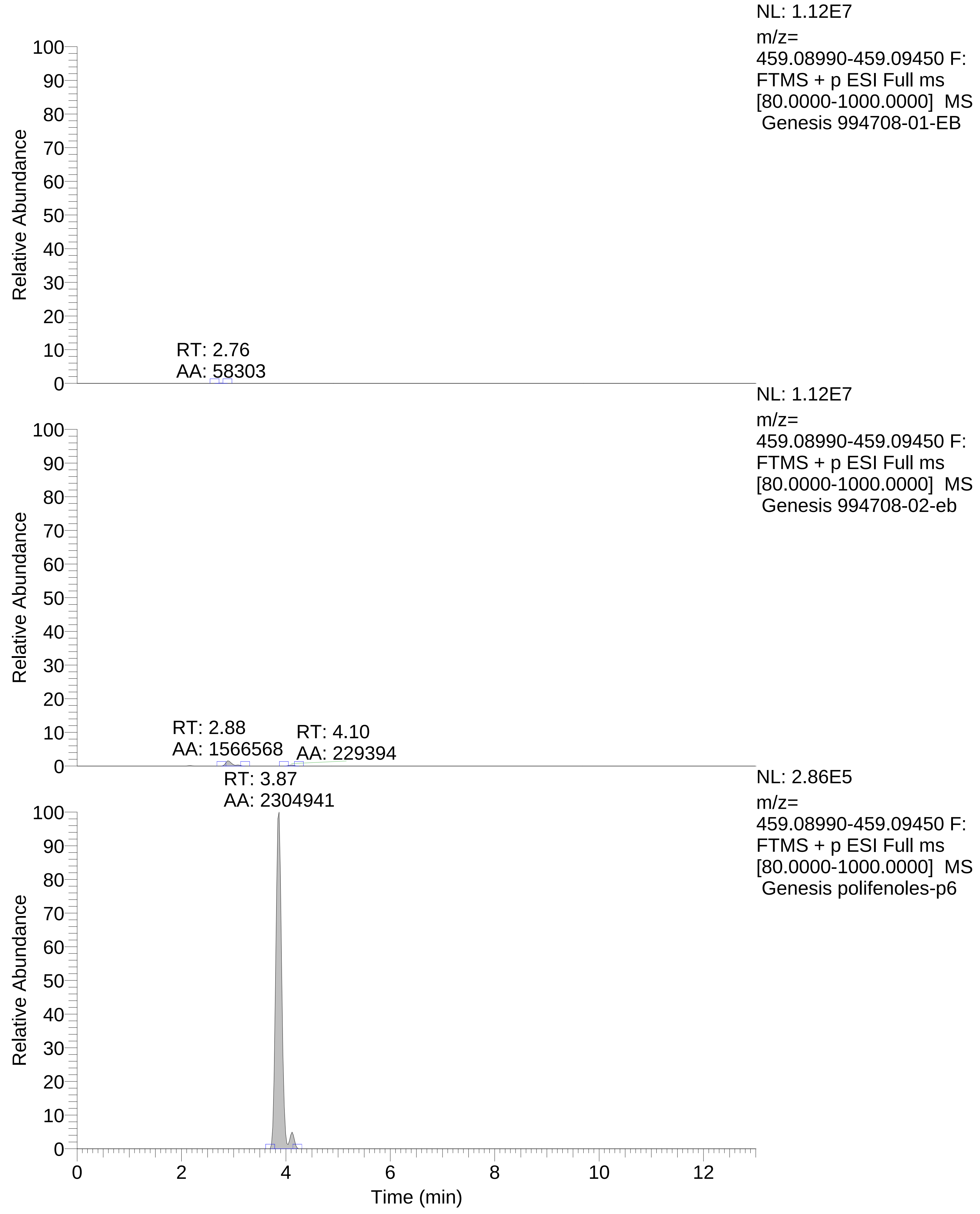

C:\Xcalibur\...\241010AG\994708-01-EB

RT: 0.00 - 13.01 SM: 9G

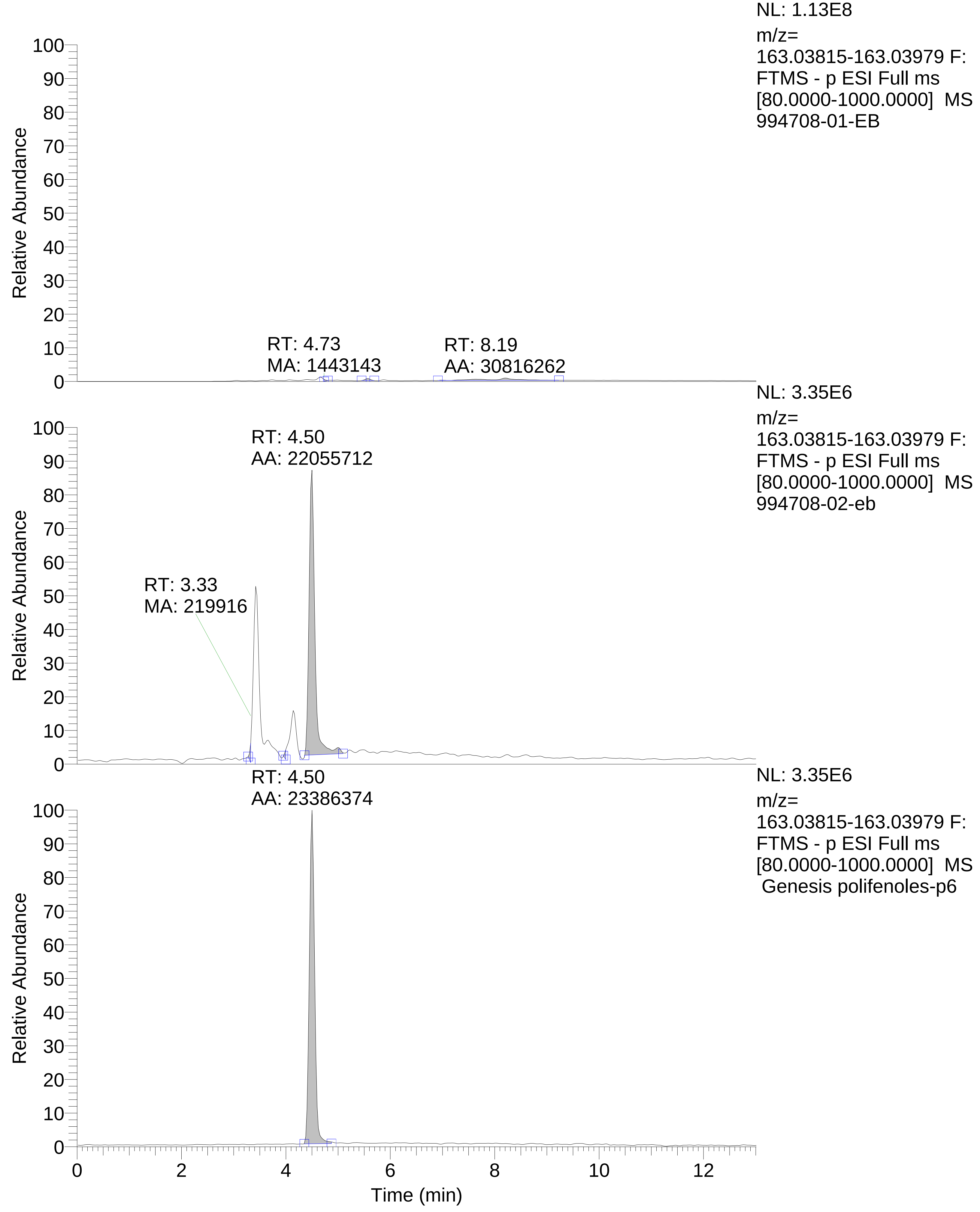

RT: 0.00 - 13.01 SM: 9G

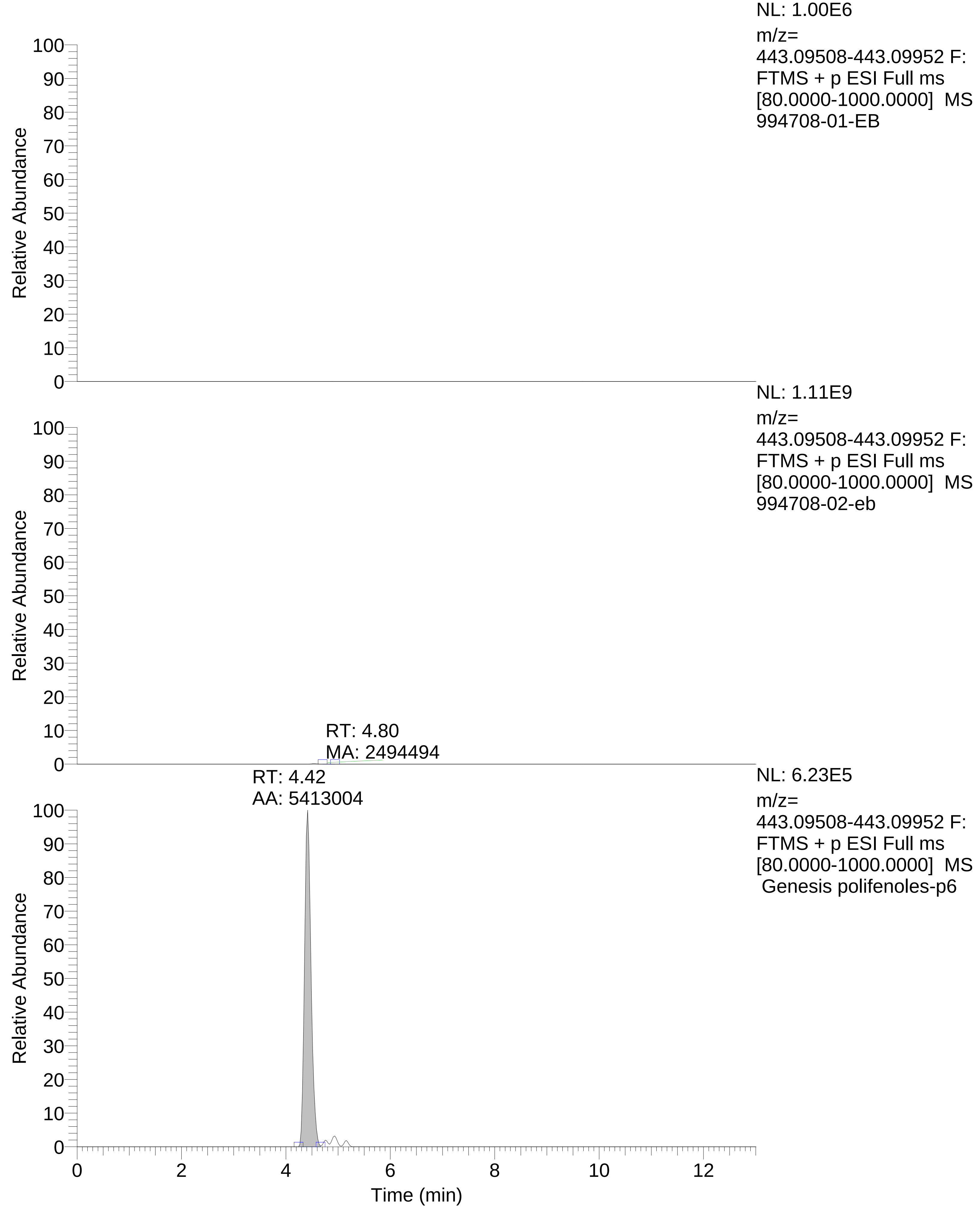

c:\xcalibur\...\241010ag\994708-02-eb

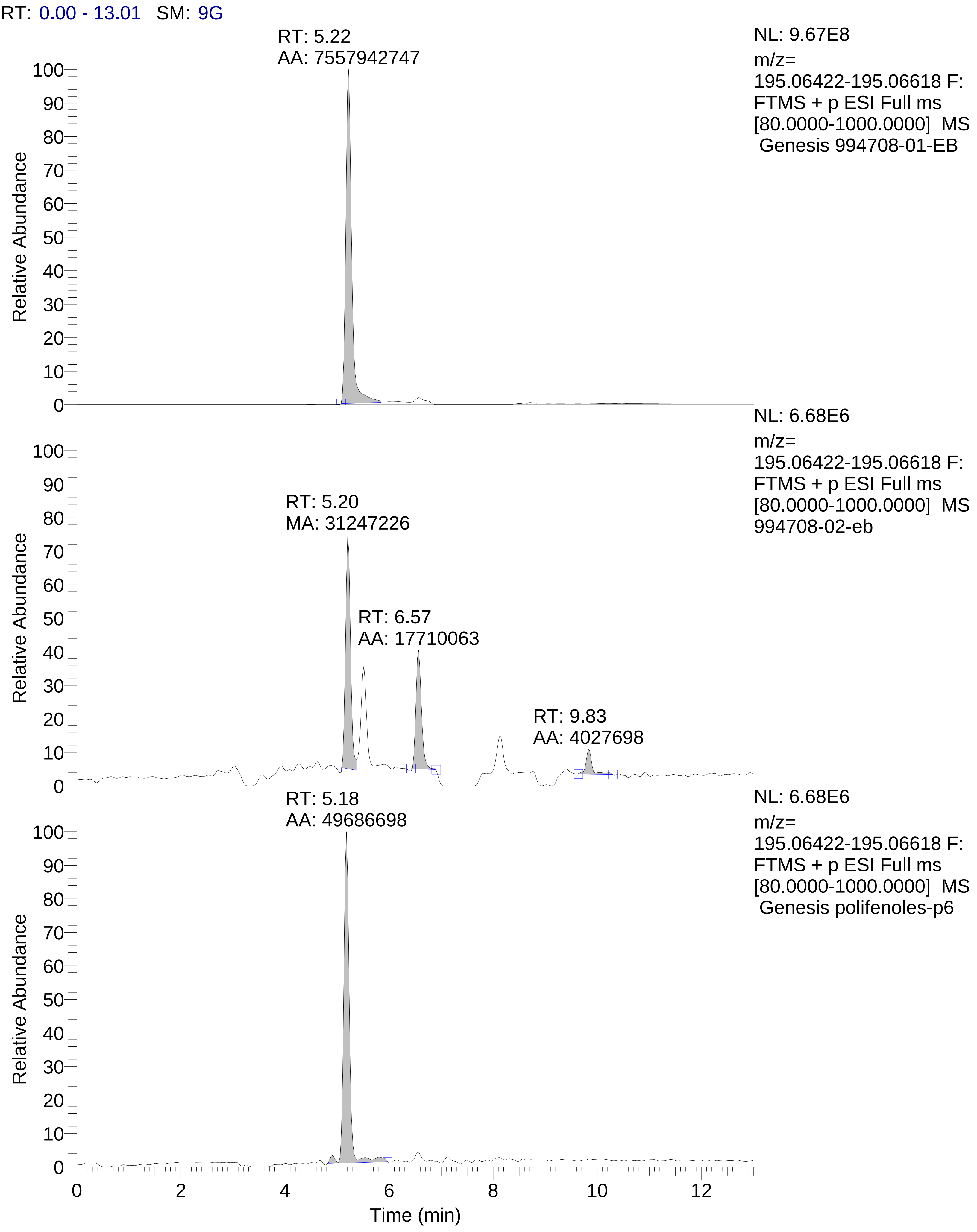

c:\xcalibur\...\241010ag\polifenoles-p6

RT: 0.00 - 13.01 SM: 9G

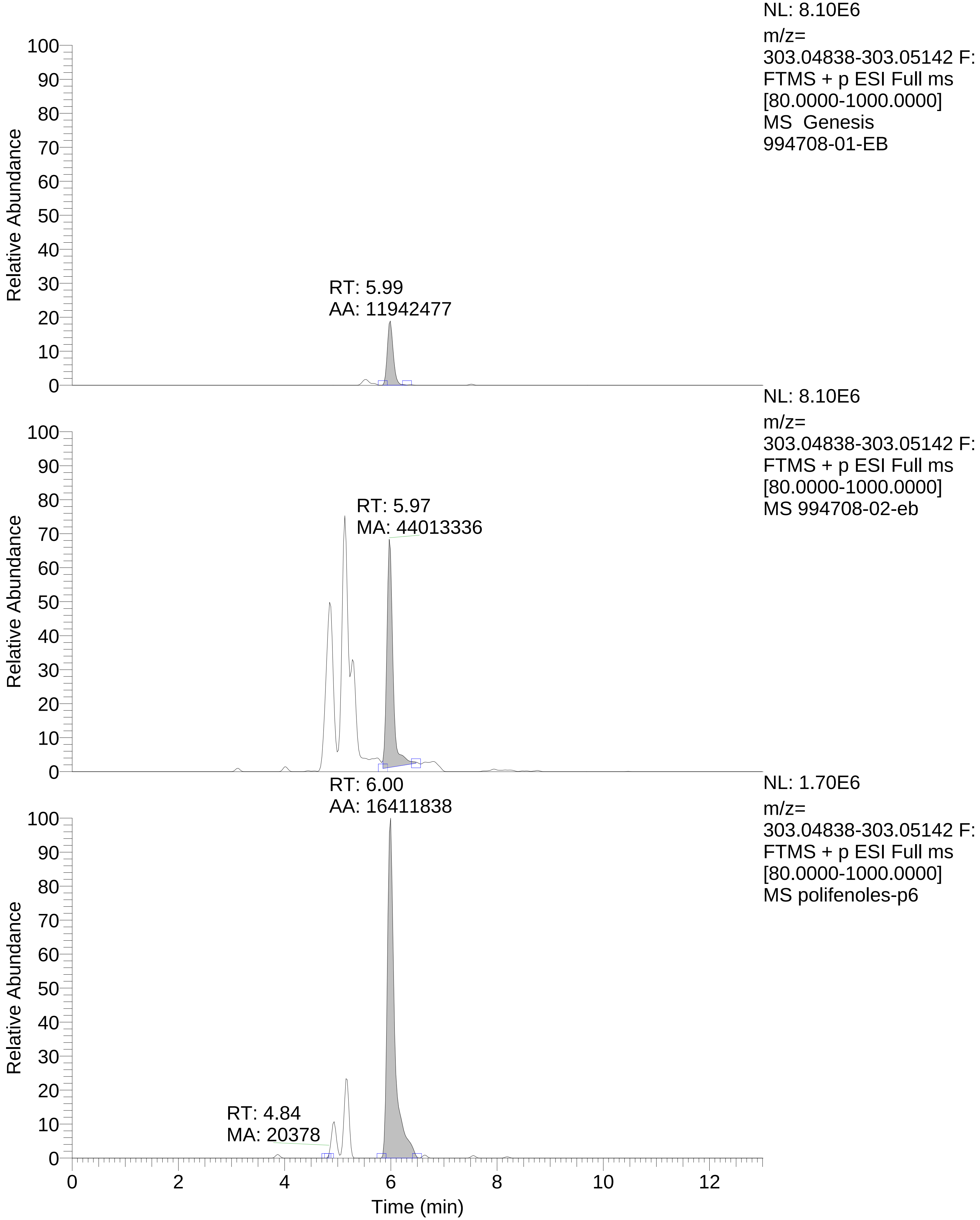

C:\Xcalibur\...\241010AG\994708-01-EB

RT: 0.00 - 13.01 SM: 9G

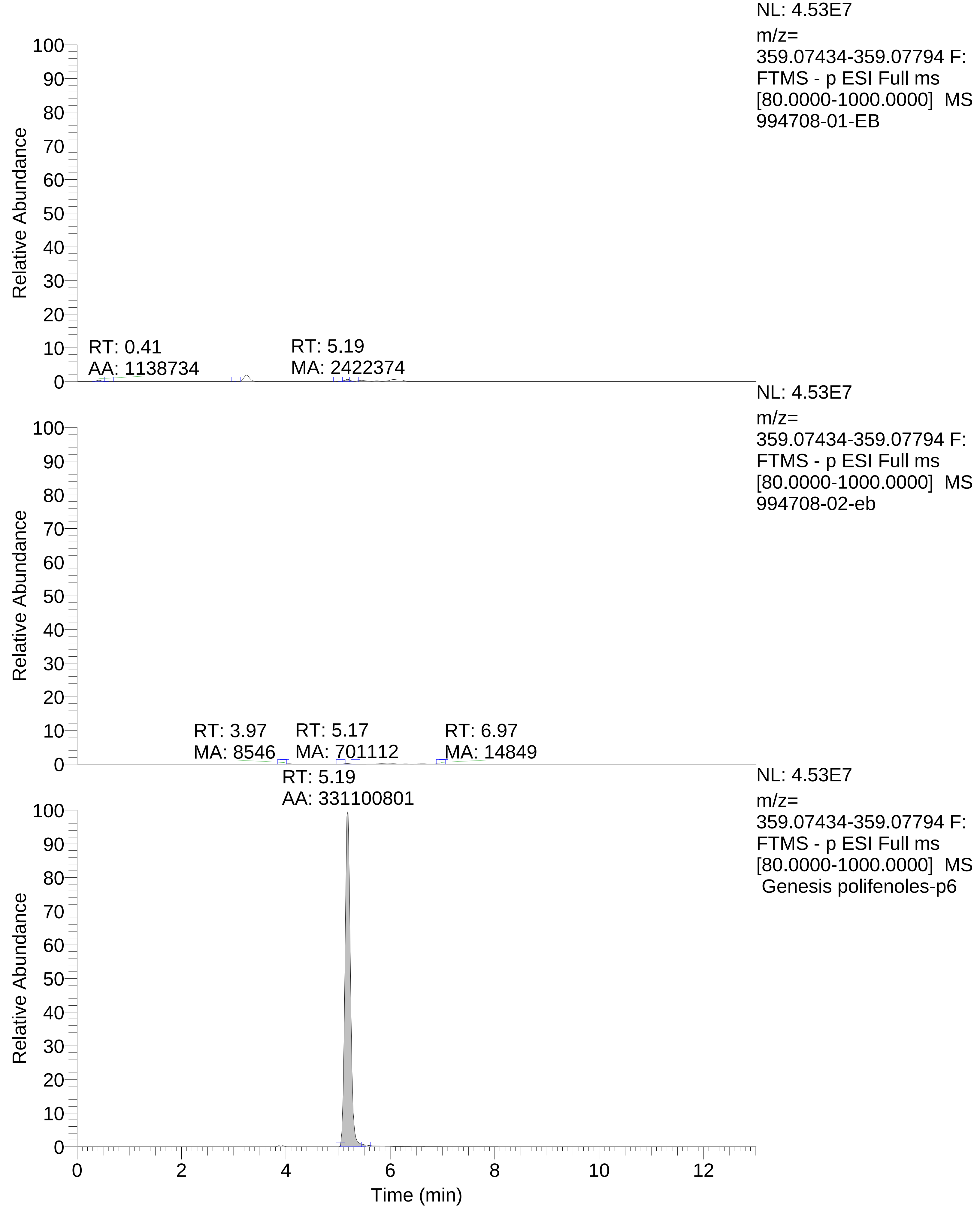

C:\Xcalibur\...\241010AG\994708-01-EB

RT: 0.94 - 5.92 SM: 9G

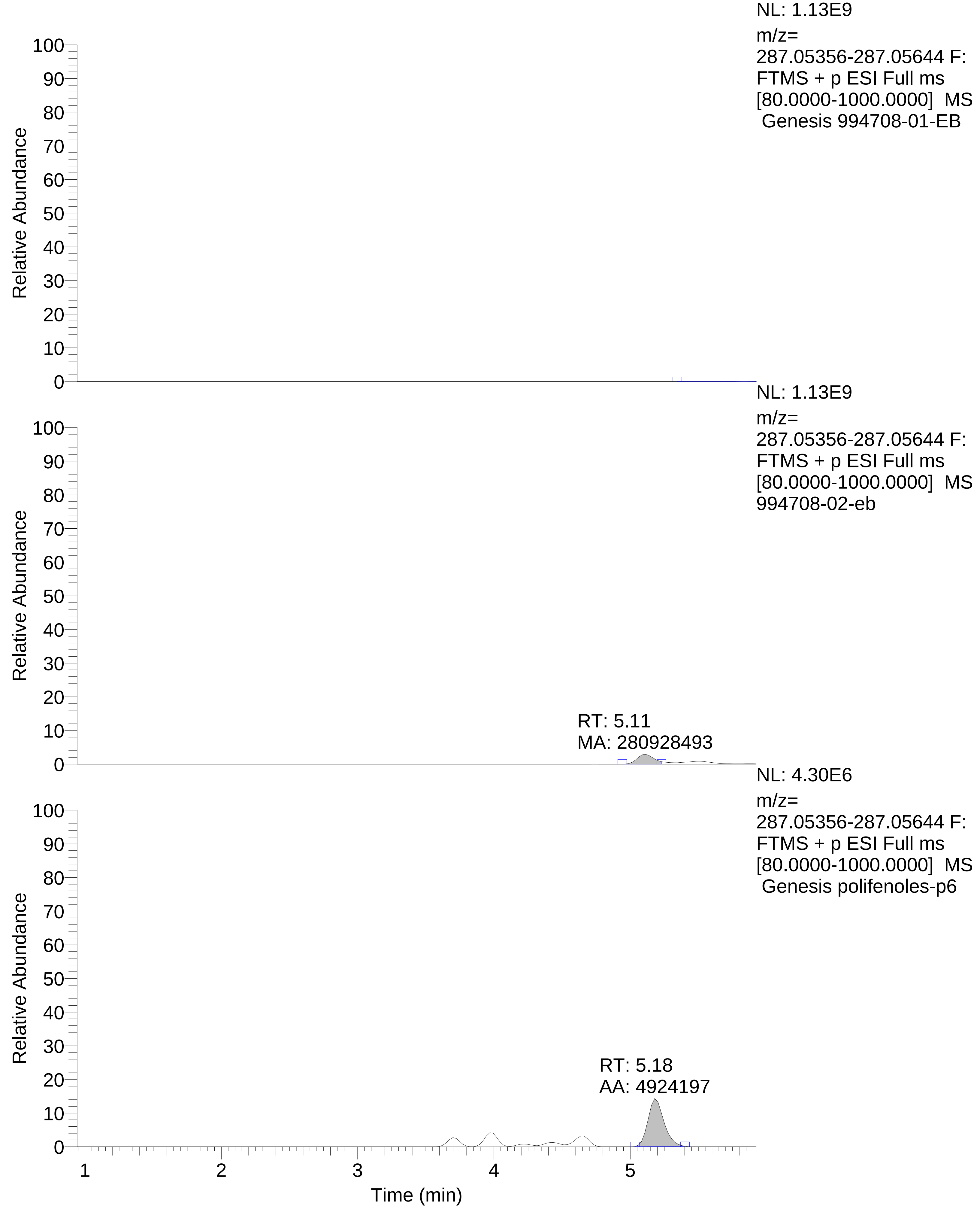

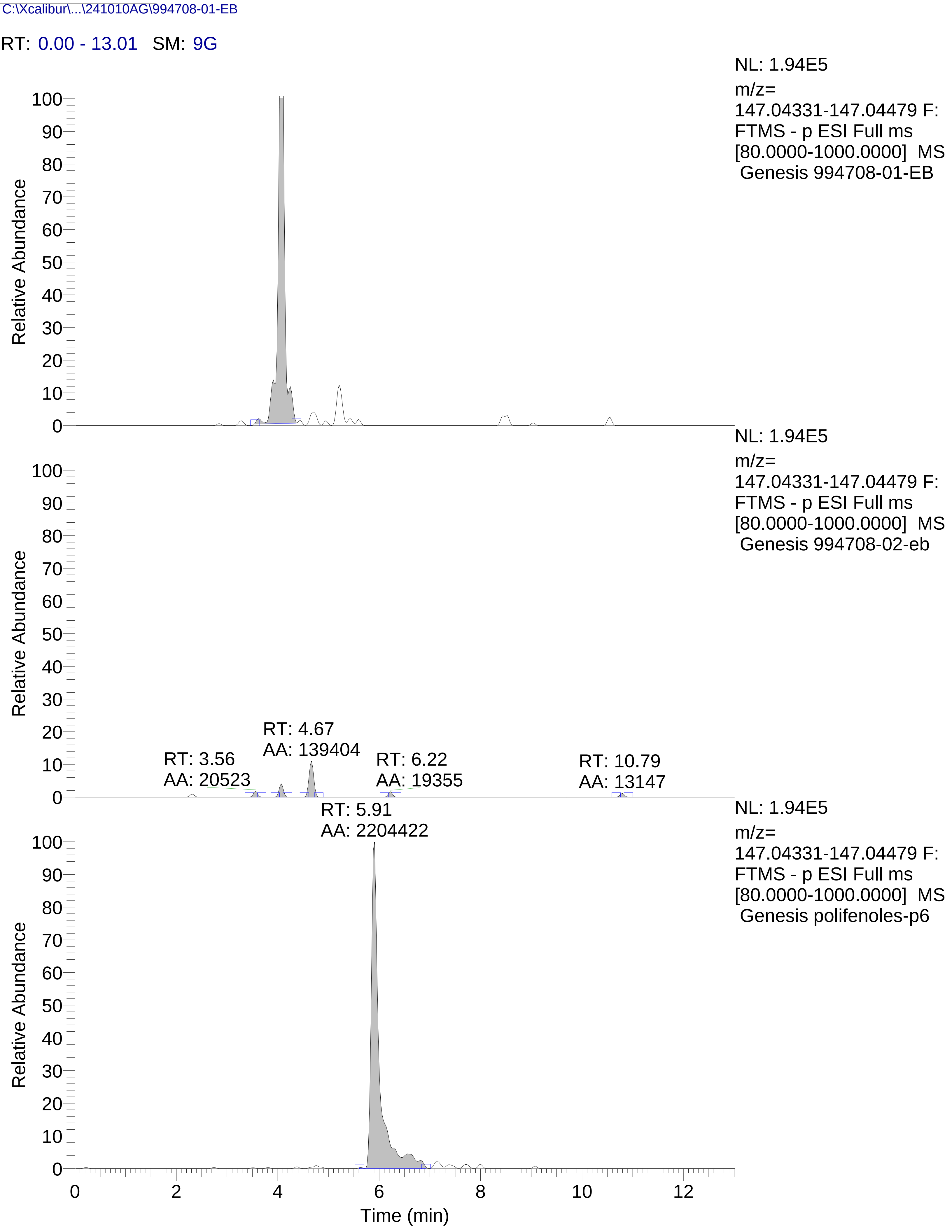
C:\Xcalibur\...\241010AG\994708-01-EB
RT: 0.00 - 13.01 SM: 9G
NL: 1.94E5
m/z= 147.04331-147.04479 F: FTMS - p ESI Full ms [80.0000-1000.0000] MS Genesis 994708-01-EB
NL: 1.94E5
m/z= 147.04331-147.04479 F: FTMS - p ESI Full ms [80.0000-1000.0000] MS Genesis 994708-02-eb
RT: 3.56 AA: 20523
RT: 4.67 AA: 139404
RT: 6.22 AA: 19355
RT: 10.79 AA: 13147
RT: 5.91 AA: 2204422
NL: 1.94E5
m/z= 147.04331-147.04479 F: FTMS - p ESI Full ms [80.0000-1000.0000] MS Genesis polifenoles-p6
Relative Abundance
Time (min)

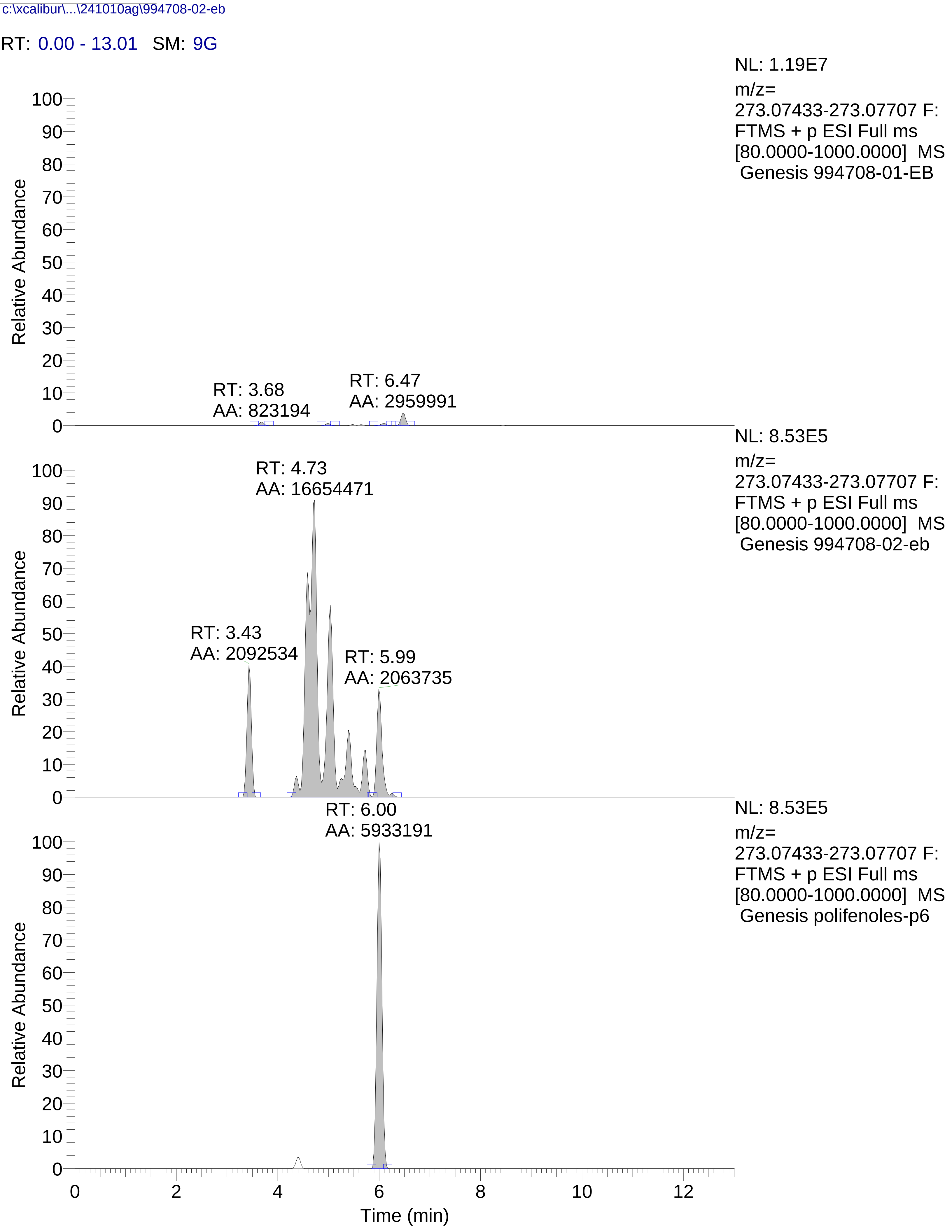
c:\xcalibur\...\241010ag\994708-02-eb
RT: 0.00 - 13.01 SM: 9G
NL: 1.19E7
m/z= 273.07433-273.07707 F: FTMS + p ESI Full ms [80.0000-1000.0000] MS Genesis 994708-01-EB
RT: 3.68
AA: 823194
RT: 6.47
AA: 2959991
NL: 8.53E5
m/z= 273.07433-273.07707 F: FTMS + p ESI Full ms [80.0000-1000.0000] MS Genesis 994708-02-eb
RT: 4.73
AA: 16654471
RT: 3.43
AA: 2092534
RT: 5.99
AA: 2063735
NL: 8.53E5
m/z= 273.07433-273.07707 F: FTMS + p ESI Full ms [80.0000-1000.0000] MS Genesis polifenoles-p6
RT: 6.00
AA: 5933191
Relative Abundance
Time (min)

c:\xcalibur\...\241010ag\994708-02-eb

RT: 0.00 - 13.01 SM: 9G

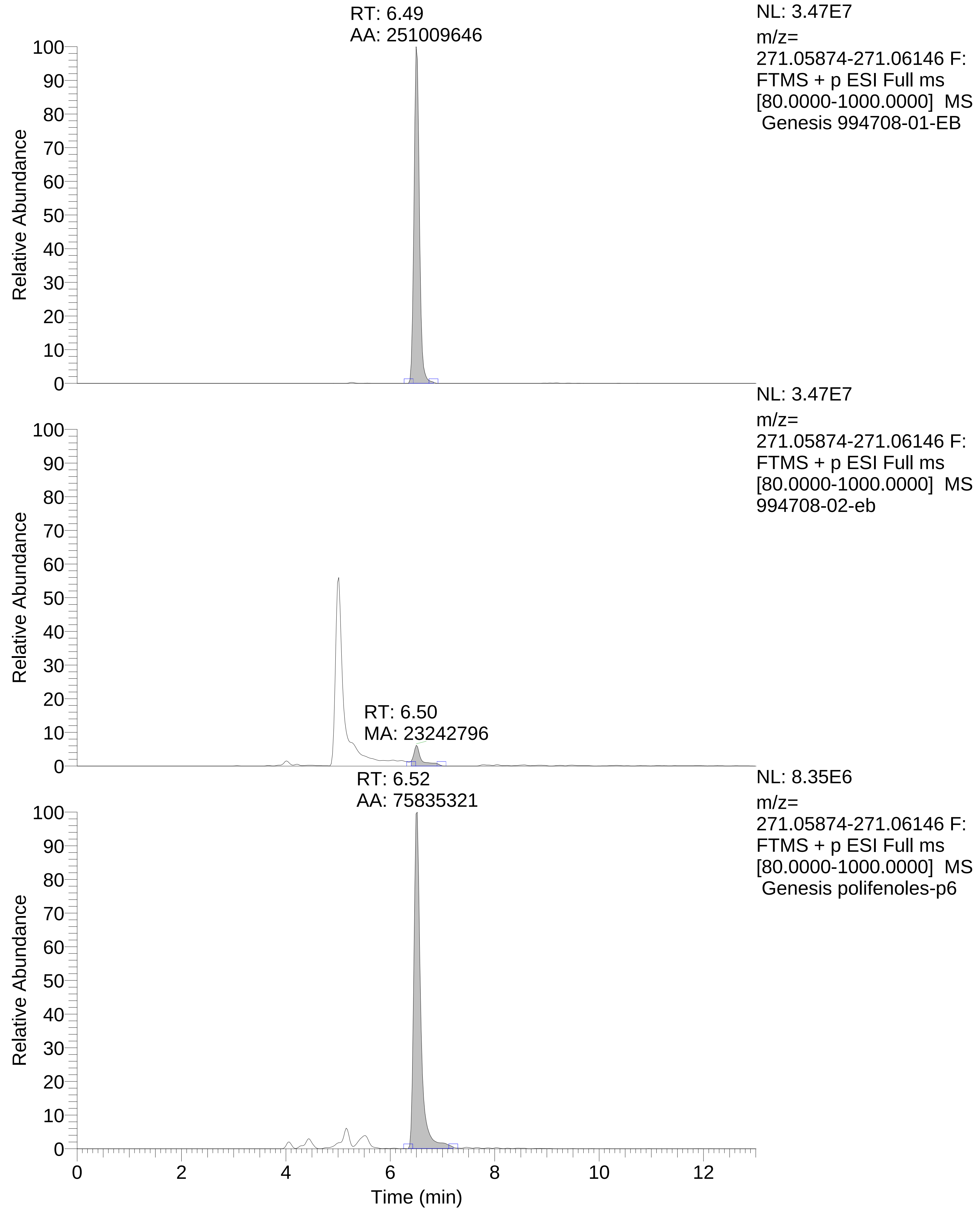

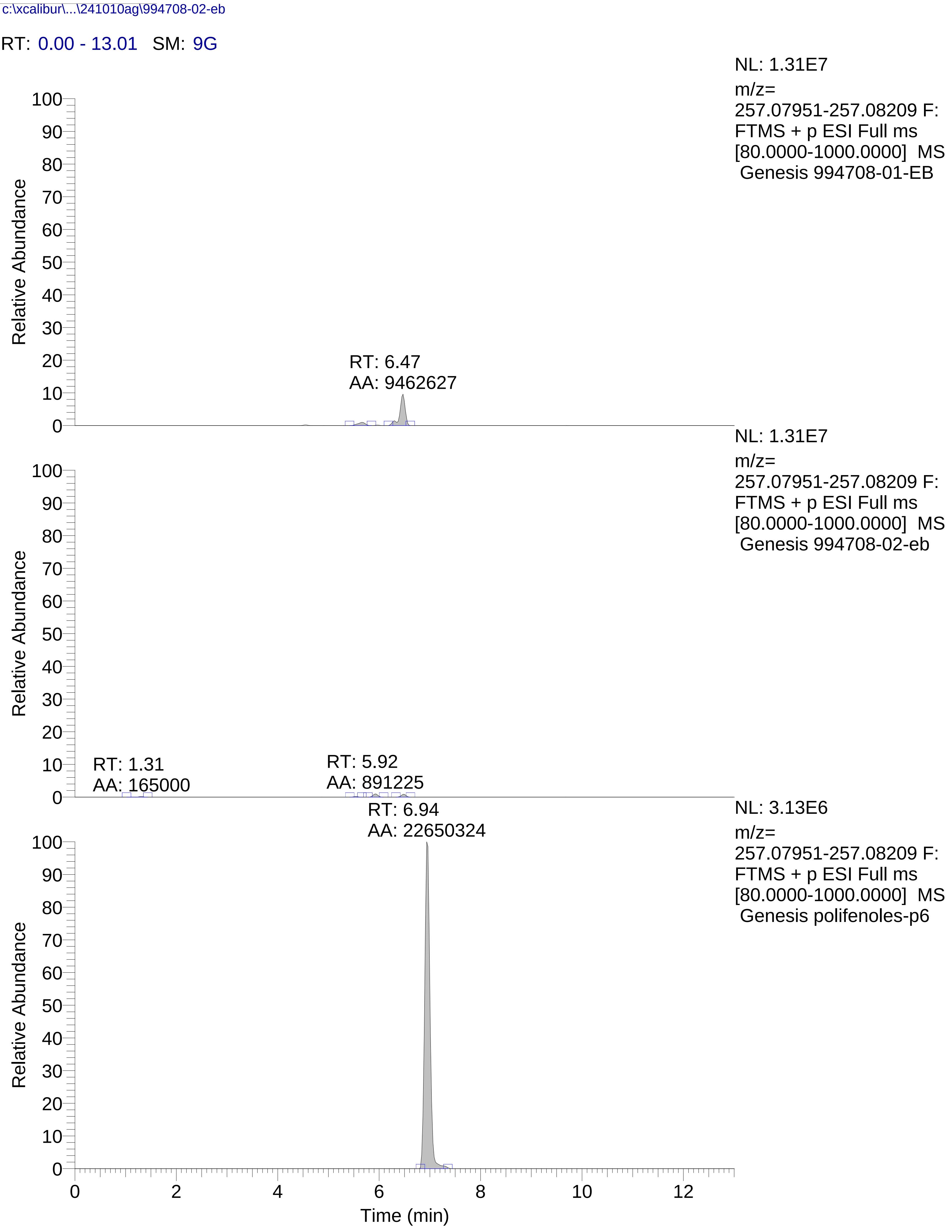
c:\xcalibur\...\241010ag\994708-02-eb
RT: 0.00 - 13.01 SM: 9G
NL: 1.31E7
m/z= 257.07951-257.08209 F: FTMS + p ESI Full ms [80.0000-1000.0000] MS Genesis 994708-01-EB
RT: 6.47
AA: 9462627
NL: 1.31E7
m/z= 257.07951-257.08209 F: FTMS + p ESI Full ms [80.0000-1000.0000] MS Genesis 994708-02-eb
RT: 1.31
AA: 165000
RT: 5.92
AA: 891225
NL: 3.13E6
m/z= 257.07951-257.08209 F: FTMS + p ESI Full ms [80.0000-1000.0000] MS Genesis polifenoles-p6
RT: 6.94
AA: 22650324
Relative Abundance
Time (min)

C:\Xcalibur\...\241010AG\994708-01-EB

RT: 0.00 - 13.01 SM: 9G

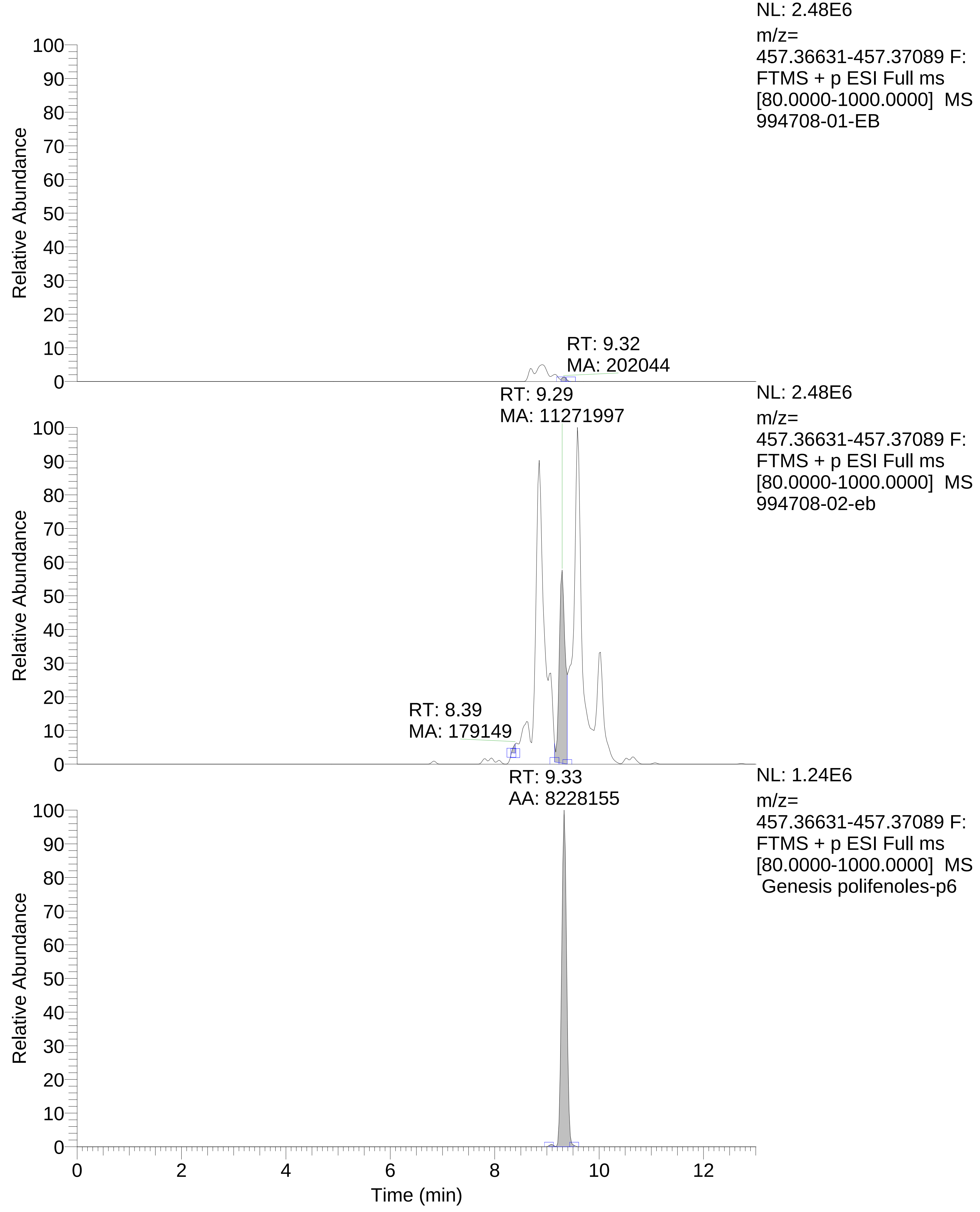

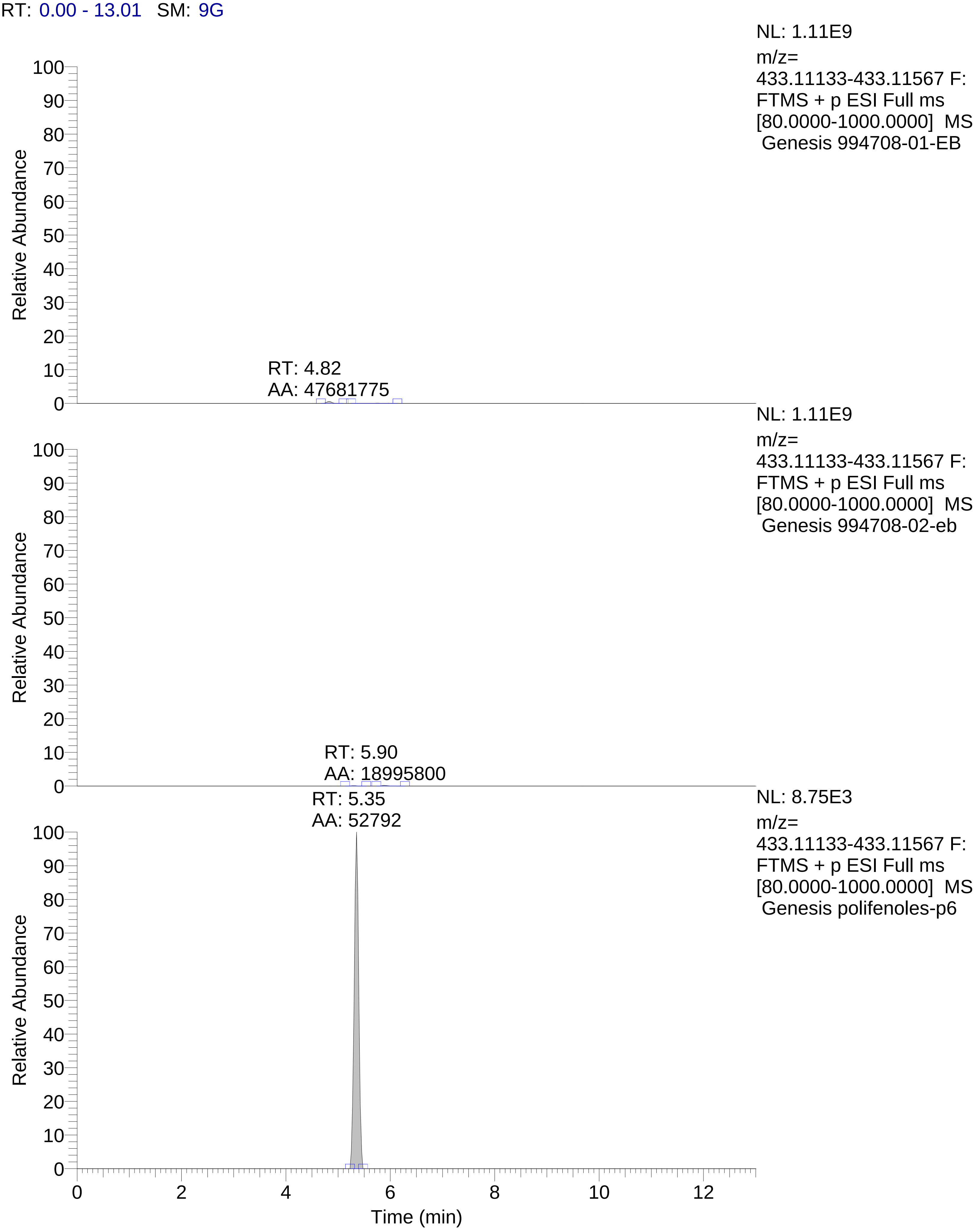
RT: 0.00 - 13.01 SM: 9G
NL: 1.11E9
m/z=
433.11133-433.11567 F:
FTMS + p ESI Full ms
[80.0000-1000.0000] MS
Genesis 994708-01-EB
RT: 4.82
AA: 47681775
NL: 1.11E9
m/z=
433.11133-433.11567 F:
FTMS + p ESI Full ms
[80.0000-1000.0000] MS
Genesis 994708-02-eb
RT: 5.90
AA: 18995800
NL: 8.75E3
m/z=
433.11133-433.11567 F:
FTMS + p ESI Full ms
[80.0000-1000.0000] MS
Genesis polifenoles-p6
RT: 5.35
AA: 52792
Relative Abundance
0
10
20
30
40
50
60
70
80
90
100
0
2
4
6
8
10
12
Time (min)

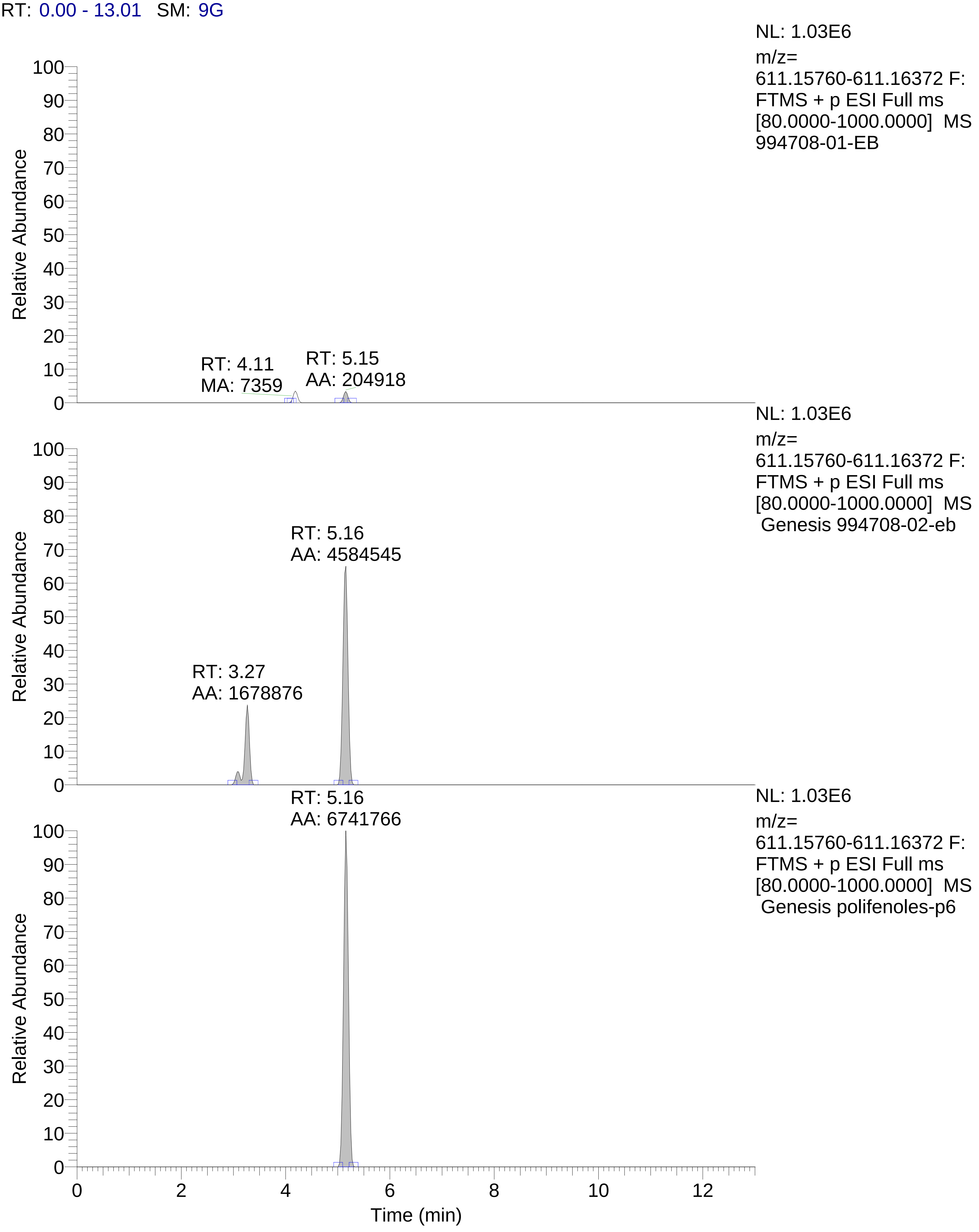
RT: 0.00 - 13.01 SM: 9G
NL: 1.03E6
m/z= 611.15760-611.16372 F: FTMS + p ESI Full ms [80.0000-1000.0000] MS 994708-01-EB
RT: 4.11
MA: 7359
RT: 5.15
AA: 204918
NL: 1.03E6
m/z= 611.15760-611.16372 F: FTMS + p ESI Full ms [80.0000-1000.0000] MS Genesis 994708-02-eb
RT: 5.16
AA: 4584545
RT: 3.27
AA: 1678876
RT: 5.16
AA: 6741766
NL: 1.03E6
m/z= 611.15760-611.16372 F: FTMS + p ESI Full ms [80.0000-1000.0000] MS Genesis polifenoles-p6
Relative Abundance
Time (min)
